# Mathematical model of hormone sensitive prostate cancer treatment using leuprolide: A small step towards personalization

**DOI:** 10.1371/journal.pone.0263648

**Published:** 2022-02-15

**Authors:** Urszula Foryś, Alon Nahshony, Moran Elishmereni

**Affiliations:** 1 Faculty of Mathematics, Informatics and Mechanics, University of Warsaw, Warsaw, Poland; 2 Institute for Medical Biomathematics, Bene Ataroth, Israel; Universite Cote d’Azur, FRANCE

## Abstract

In this paper we present a new version of a mathematical model of Elishmereni et al. describing androgen deprivation therapy (ADT) for hormone sensitive prostate cancer patients (HSPC). We first focus on the detail description of the model, and then we present mathematical analysis of the proposed model, starting from the simplified model without resistance and ending on the full model with two resistance mechanisms present. We make a step towards personalization proposing an underlying tumor growth law base on a cohort of patients from Mayo hospital. We conclude that the model is able to reflect reality, that is in clinical scenarios the level of testosterone in HSPC patients inevitably rises leading to the failure of ADT.

## Introduction

Prostate cancer is the second most common cancer worldwide among men, and incidence rates are increasing every year [1]. Hormone sensitive prostate cancer (HSPC) patients are treated by androgen deprivation therapy (ADT) as the standard of care, applied either continuously or intermittently [2]. Eventually, however, androgen-independence emerges and the disease progresses to the most advanced stage of prostate cancer, castrate-resistant prostate cancer (CRPC) which often occurs concomitantly with metastatic disease [3]. The clinical occurrence of this process is signaled by a surge in the tumor surrogate biomarker prostate specific antigen (PSA) while under ADT, otherwise termed biochemical failure on ADT [4].

The time to biochemical failure (TTBF) and progression to CRPC in ADT-treated patients can span from a few months to ca. 3 years [5]. Anticipating this time in the individual patient can have a vast impact on effective treatment planning and ultimate clinical outcome [6], yet TTBF remains very hard to predict. Some biomarkers and prognostic factors have been suggested based on statistical studies, yet none have reached clinical significance [7]. The large variability in tumor PSA profiles among patients also gravely complicates the prediction of the patient’s TTBF.

Mathematical modeling of tumor growth has a long history, starting from early papers of A.K. Laird [8, 9]. However, these early papers were related to some general description, not focusing on specific laws for specific tumors. On the other hand, such general growth laws are still used. In [10] the reader can find a thorough review on the models used in mathematical modeling of prostate cancer growth and various types of treatment. Specific model, addressing the problem of ADT for HSPC patients, we would like to consider in this paper, was proposed in 2016 by Elishmereni et al. [11]. In [11] Elishmereni and colleagues published their work on a personalized algorithm for predicting TTBF in HSPC patients. This algorithm was based on a dynamic mathematical model and was trained on a real-world patient database from a hospital registry. Here, we expand this model and refine it to add a more mechanistic biological backbone, and include pharmacokinetics/pharmacodynamics (PK/PD) of one ADT agent, leuprolide. Efforts are also made toward decreasing the inter-individual variability and the number of patient-specific parameters in the new model.

## Methods

### Scheme of the new model

**Fig 1:**
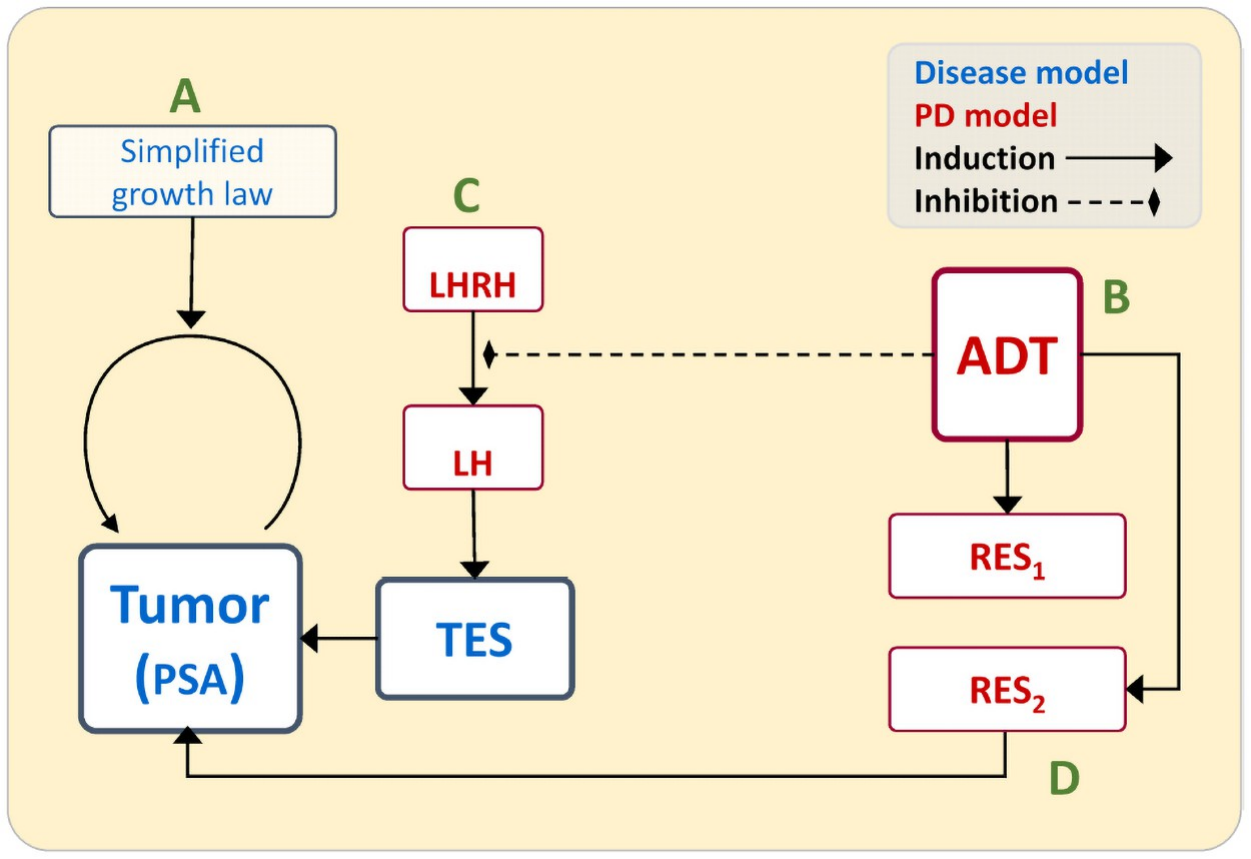
Schematic representation of the model of the ADT therapy for HSPC patients.

In this subsection we focus on the description of the model of ADT therapy for HSPC patients. Schematic diagram of the model is presented in Fig 1.

As it could be seen in the diagram first (cf. panel A in Fig 1), we would like to propose a simplified law reflecting the underlying tumor growth. We assume that this should be an equation reflecting changes in prostate specific antigen (PSA) level which we take as a measure of the tumor size. We base on a general equation of the form $\dot{P}=Pf\left(P\right)$, where *P*(*t*) is the level of PSA at time *t*, while *f* is a positive function which reflect the per capita growth rate and will be specified later on the basis of patients data.

Second (panel B in Fig 1), we need to describe PK/PD of the drug. Third (panel C in Fig 1), the process of testosteron production will be included, and finally (panel D) we plan to include some mechanisms of resistance. Note that there are many possible mechanisms of resistance (several different biological pathways; c.f. [12, 13]), and since we do not know which of them are more relevant here, we decided to address resistance more vaguely (as 1–2 components), fit to the phenomenology of the ultimate PSA incline indicating that resistance has occurred. This idea of including resistance comes directly from the original paper [11].

### Dataset

**Fig 2:**
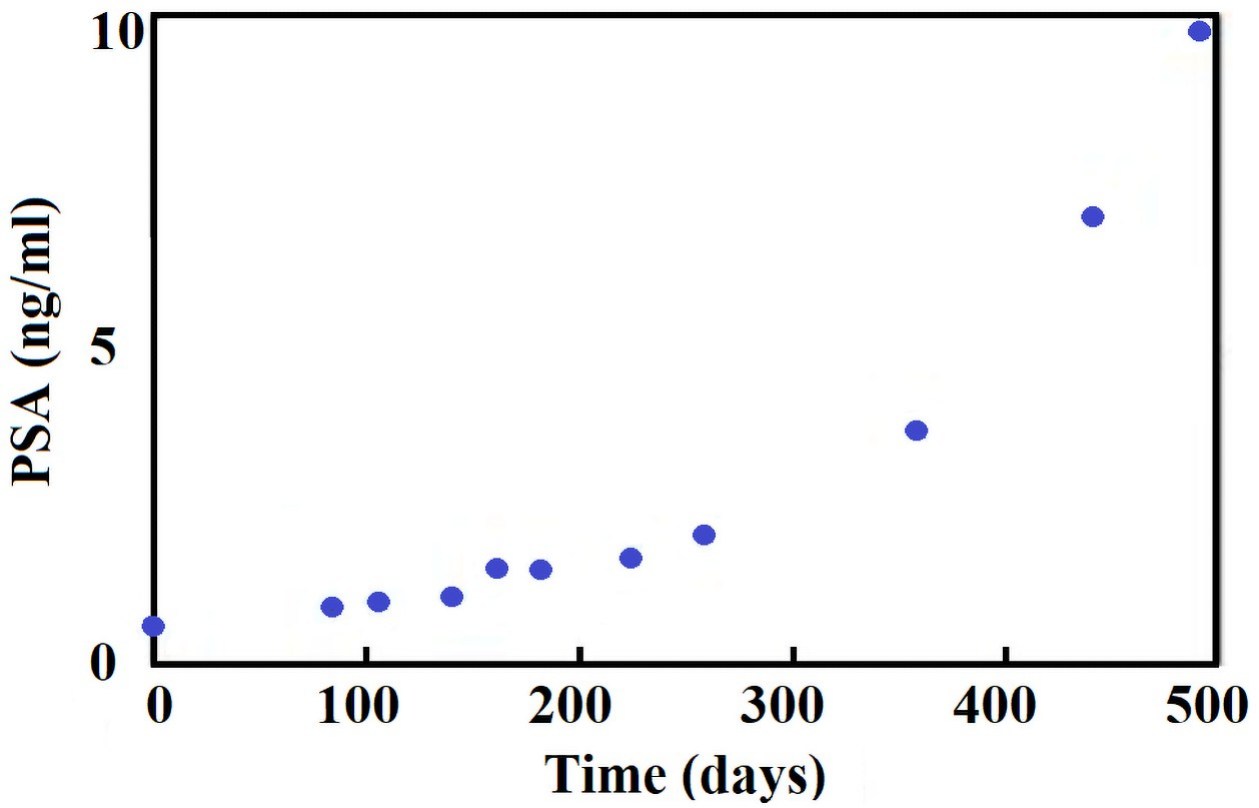
An exemplary course of PSA level (patient No. 1248) in time.

**Fig 3:**
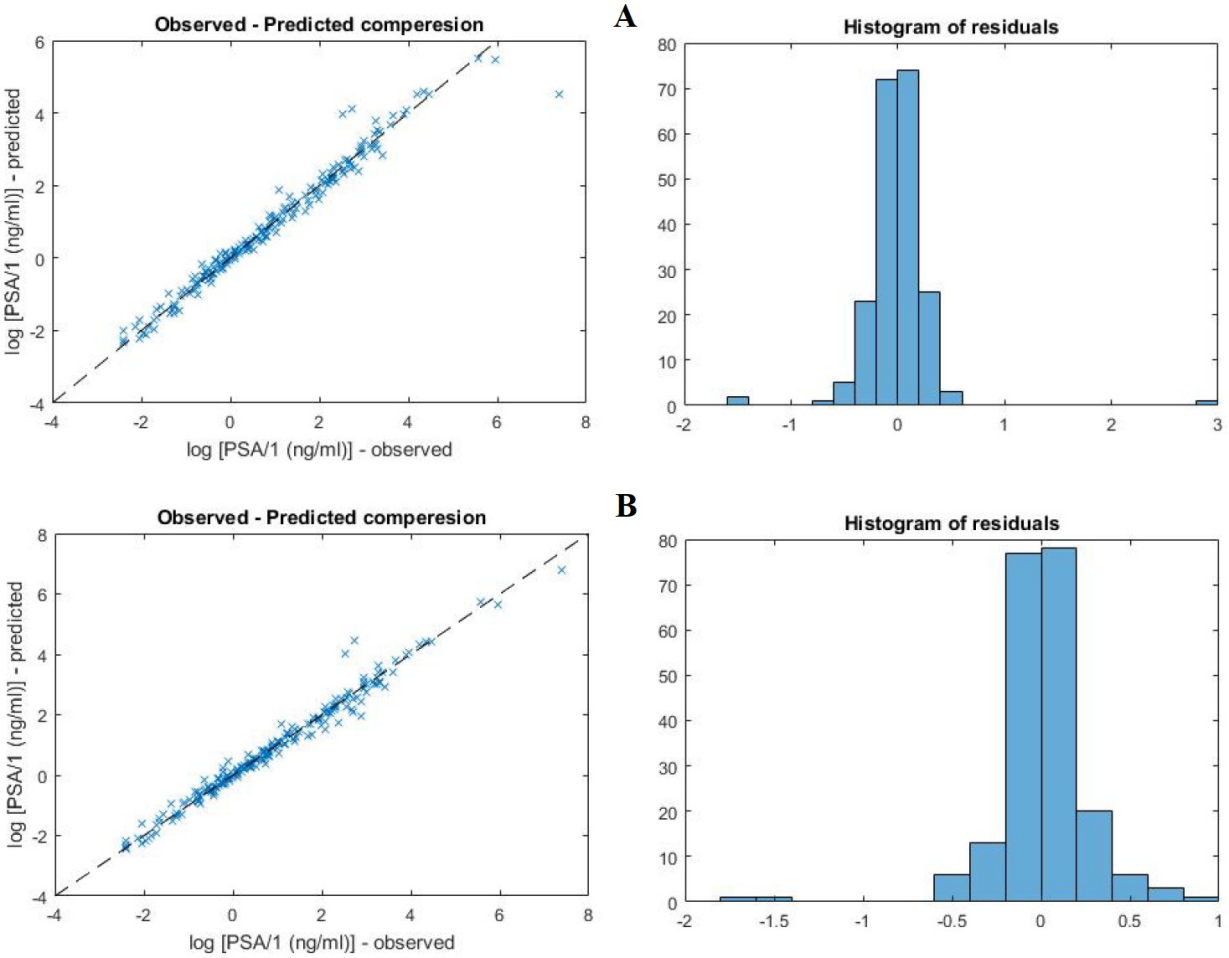
Comparison between the fits obtained for the growth law proposed in this paper (panel A) with per capita growth rate described by Eq (2) and the model proposed in [11] (panel B).

In this study we use some part of the dataset described in more details in the original paper [11]; cf. Table 1 at page 3 for statistical summary of the whole dataset, Fig 1 at page 3 for a typical structure of the data for one patient and Fig 3 at page 6 for several examples of specific patients data. Here, in order to propose a simplified growth law of the tumor, we constructed a subdataset of patients with PSA dynamics before the first application of ADT. This dataset comprises 206 data points and total of 19 patients, and is summarized in Table 1. An exemplary course of PSA level in time for one of the patients is presented in Fig 2.

**Table 1:** Summary of the pre-treatment dataset.

property	median	range
# of data points per patient	10	6–22
timespan (days)	1162	388–5724
PSA (ng/ml)	2	< 0.1–1600

### Nonlinear mixed effect modeling

Fitting parameters of assumed model to the data we use a statistical method called in general mixed effect modeling. Here we focus on the nonlinear method (NMEM); cf. e.g. [14] for detailed description. In this method, for *M* individuals numbered by *i* = 1…*M* we have *n*_*i*_ measurements. If *y*_*ij*_ denotes the *j*th observation for *i*th individual, then we assume

$$\begin{array}{c} y_{ij}=f\left(\phi_{ij},x_{ij}\right)+\varepsilon_{ij},\;i=1\ldots M,\;j=1,\ldots n_{i}. \end{array}$$

Here *f* is a nonlinear function that depends on some parameters *ϕ*_*ij*_ and predictions *x*_*ij*_, and *ε* reflects normally distributed noise. Furthermore, for parameters *ϕ*_*ij*_ we assume

$$\begin{array}{c} \phi_{ij}=A_{ij}\beta +B_{ij}b_{i},\;b_{i}∼N\left(0,\sigma^{2}D\right), \end{array}$$

where *β* describes so-called fixed effect (i.e. parameters fixed for the population), *b*_*i*_ describes so-called random effect (i.e. parameters varying randomly between individuals), *A*_*ij*_, *B*_*ij*_ are so-called design matrices for these types of effects, respectively, and *σ*^2^ *D* is a variance-covariance matrix. Parameters are estimated using maximum likelihood; cf. [14].

In our application of this method we did not include any covariates. We estimated the individual parameters from the patients PSA data, as the modes of posterior distributions. Hence the design matrix is just constant. The betas are the means of the individual parameters.

## Description of the model

This section is devoted to the detailed description of the proposed model introduced in Fig 1.

### Underlying tumor growth

As mentioned above, to reflect tumor size we use the level of prostate specific antigen (PSA) denoted by *P*(*t*). In general, we would like to explore the growth law described in a simple manner by the equation

$$\begin{array}{c} \dot{P}=Pf\left(P\right), \end{array}$$ (1)

where the *per capita* growth rate $f:R^{+}\to R^{+}$ is a locally Lipschitz continuous function, guaranteeing existence of unique solution for any nonnegative initial data $P_{0}\in R^{+}$. To specify the function *f* we explored some of the well known functions used in the literature (cf. e.g. [15] for the description of various growth functions). Following the ideas of A.K. Laird [8, 9], many researchers use Gompertz [16, 17] model with $f\left(P\right)=-a\;\text{ln}\;\frac{P}{K}$ to describe tumor growth. Here *a* reflects maximal tumor growth rate and *K* is maximal tumor size, known as carrying capacity in ecology. Another well known model is the logistic equation proposed by P.F. Verhulst [18], for which $f\left(P\right)=a(1-\frac{P}{K})$, with the same meaning of parameters. Although this model has simple mathematical structure, it is considered as not so well grounded in biology (cf. [15] and also [19] for the discussion on that topic). Yet another possibility is to add additional parameter reflecting spatial relations, like in the Greenspan [20–22] model, which is a special case of generalized logistic equation, $f\left(P\right)=a(1-{\left(\frac{P}{K}\right)}^{\gamma })$ with *γ* = 2/3. All the mentioned models have similar qualitative properties: the growth for small *P* is fast, next there is an inflection point and the growth saturates at the carrying capacity *K*. However, in the case we are interested in, we need to describe the PSA level only on finite interval, for which the ADT therapy is used, which means that the asymptotic behavior of PSA is not important. Hence, the function *f* needs not lead to the saturated growth. Therefore, the simplest growth law that could be used is just exponential, with constant *f*. The growth law proposed in [11] includes modulated exponent, that is $\dot{P}=a{\left(\frac{P}{P_{R}}\right)}^{k(t)}$, where *k* increases linearly with time, i.e. $\dot{k}=\lambda$, and *P*_*R*_ > 0 is some constant level of PSA.

Specific form of the function *f* we used is related to the nature of the patients data. For 19 of the patients the procedure of watchful waiting was applied, so that for them we have data showing that without the treatment the growth is faster than exponential, at least for the part just before starting the ADT treatment. To fit the model to the data we used nonlinear mixed effect modeling (NMEM) method (cf. e.g. [14] for NMEM description) implemented in MATLAB.

We made a fitting for several models described above and listed in Table 2. More precisely, for all the presented models, using NMEM we fitted $\text{log}\frac{P(t)}{P_{R}}$ (where *P*_*R*_ is some reference value of PSA which is constant and not considered as additional parameter) to the logarithms of the PSA values in patients data. In general, as could be expected and seen in Table 2, the more parameters the model has, the better fit could be obtained. However, this is not always the case. Clearly, the exponential model with only one parameter is better fitted than the Gompertz model with two parameters. This is related to the idea upon which the Gompertz model was built, that the tumor growth is much faster than exponential at the early beginning. However, it seems that for the case we consider this assumption is not valid. Moreover, better fit is obtained for models with the growth faster than exponential at the and of observed interval.

**Table 2:** Models used for fitting and respective per capita growth rates vs statistical and information criteria describing quality of the fit.

model	*f*(*P*)	MSE	R^2^	NLL	AIC	BIC
exponential	*a*	0.4073	0.9513	183.95	379.91	399.88
Gompertz	−*a* ln(*P*/*K*)	0.4305	0.9310	197.69	409.37	415.98
logistic	*a*(1 − *P*/*K*)	0.3802	0.9479	182.65	379.29	385.90
general. logistic	*a*(1 − (*P*/*K*)^*γ*^)	0.3776	0.9495	180.69	379.37	387.87
our model	*a*(1 + *b* ln *P*/*P*_*R*_)^*γ*^	0.3138	0.9652	171.03	356.05	362.67
time modulated	*a*(1 + λ*t*)(*P*/*P*_*R*_)^*γ*^	0.3310	0.9617	173.32	364.63	373.13
original [11]	*a*(*P*/*P*_*R*_)^*k*(*t*)^	0.2673	0.9756	154.72	327.45	335.95

Here: MSE—mean square error, NLL—negative log-likelihood, AIC—Akaike Information Criterion, BIC—Bayesian Information Criterion.

Although the best fit was obtained for the original growth law proposed in [11] (cf. Table 2 and Fig 3), we decided to use simplified model with the following *per capita* growth rate:

$$\begin{array}{c} f\left(P\right)=a{\left(1+b\;\text{ln}\;\frac{P}{P_{R}}\right)}^{\gamma },\;\gamma \in \left(0,1\right), \end{array}$$ (2)

where *P*_*R*_ could be considered as the level of detection here. Our decision is mainly related to the simpler form and better mathematical properties of the model proposed above comparing to the model from [11]. Clearly, our model is described by one autonomous differential equation, while the previous model needs two such equations because the power *k*(*t*) depends on time.

Note that the function *f* defined by Eq (2) is well defined only for such values of *P* for which $1+b\;\text{ln}\;\frac{P}{P_{R}}>0$, that is for

$$\begin{array}{c} P>P_{R}{\;\text{e}}^{-1/b}=P_{c}. \end{array}$$

However, we use the model for *P* > *P*_*R*_(> *P*_*c*_), as below the level of detection we do not register the values of PSA. On the other hand, we want to have the growth law defined for all *P* ≥ 0. Hence, we extend the function *f* in a continuous manner such that *f*(*P*) = *a* for *P* ≤ *P*_*R*_, that is we have exponential growth below the level of detection. This means that instead of Eq (2) we consider

$$\begin{array}{c} f\left(P\right)=\{\begin{array}{cc} a, & \text{for}\;P\in [0,P_{R}],\\ a{\left(1+b\;\text{ln}\;\frac{P}{P_{R}}\right)}^{\gamma }, & \text{for}\;P>P_{R}, \end{array} \end{array}$$ (3)

and the growth law is described by Eqs (1) and (3).

### PK/PD model for leuprolide

The drug, whether it is leuprolide or another druf used in ADT, is typically given as a depot, which is a small implant that is injected under the patient’s skin. This provides a slow-release of the drug over a given period, allowing for continuous shut down of the hormonal cascade and therefore continuous inhibition of testosterone production (and PSA production), without the patient having to frequently visit the doctor for more medication. Hence, delayed absorption is observed. Some typical dosages include: 7.5 mg—one injection every 4 weeks, 22.5 mg—one injection every 12 weeks, 30 mg—one injection every 16 weeks, 45 mg—one injection every 24 weeks. In this section we introduce two ideas of including the influence of leuprolide into the model.

#### Mass action law based PK model

The first idea of modeling the influence of the drug comes from the paper of Lim and Salem [23], where the authors presented a semi-mechanistic model of PK/PD of leuprolide for prostate cancer patients which was fitted to patients data using NMEM. To describe PK of the drug they assumed that there are three depots of the drug: first, related to the delayed absorption, second, corresponding to the first order absorption, and third, corresponding to the zero-order absorption. The drug is supplied to the so-called central compartment and there is an exchange of leuprolide between the central and peripheral compartments. In [23] the authors mentioned that they tried to model the delayed absorption using delay differential equations but it occurred that better fitting can be obtained for considering additional transit compartments through which the drug flows before absorption in the central compartment.

Let *D*(*t*) denote the drug concentration that reaches to all the depots after an application, *L*_*c*_(*t*) and *L*_*p*_(*t*) denote concentrations of leuprolide in so-called central and peripheral compartments specific for the drug. Moreover, we have *n*-transit compartments *L*_*i*_, *i* = 1, …, *n* serving as a delayed absorption of leuprolide. Then, basing on the scheme of PK model presented in [23], the described PK model could be written as

$$\begin{array}{c} \begin{array}{cc} \dot{D} & =-\beta D,\\ {\dot{L}}_{1} & =k_{tr}D-k_{tr}L_{1},\\ {\dot{L}}_{2} & =k_{tr}L_{1}-k_{tr}L_{2},\\ \vdots & =\vdots ,\\ {\dot{L}}_{n} & =k_{tr}L_{n-1}-k_{tr}L_{n},\\ {\dot{L}}_{c} & =k_{1}L_{n}+k_{2}D+k_{3}\chi \left(t\right)-QL_{c}+QL_{p}-d_{L}L_{c},\\ {\dot{L}}_{p} & =QL_{c}-QL_{p}, \end{array} \end{array}$$ (4)

where *β* is the drug clearance rate, *k*_*tr*_ are rates of transition along the delayed path, *k*_1_, *k*_2_ are coefficients for first-order delayed and immediate absorptions, *k*_3_ reflects zero-order absorption, *χ*(*t*) = 1 during the ADT infusion and *χ*(*t*) = 0 otherwise, *Q* describes the flux between central and peripheral compartments and *d*_*L*_ is a clearance rate from central compartment.

It seems that we can simplify (4) even more, as if the compartments *L*_*i*_ serve just as a transition of the drug then assumptions *β* = *k*_*tr*_ and *k*_*tr*_ = *k*_1_ are not pointless. It is obvious that knowing the ADT application scheme we are able to solve this system of linear equations.

Assuming one application of the drug we obtain $D\left(t\right)=\alpha {\text{e}}^{-k_{1}t}$, where *α* is related to the portion of the drug absorbed via delayed path, and next we calculate (for details see A Appendix in S1 Appendix)

$$\begin{array}{c} L_{j}\left(t\right)=\alpha \frac{k_{1}^{j}t^{j}}{j!}{\text{e}}^{-k_{1}t}, \end{array}$$

which then reflects a kind of distribution of the drug in the *L*_*c*_–*L*_*p*_ system. Note that for single input of *ADT* we can omit zero-order absorption, so we assume *k*_3_ = 0. Moreover, we realized that using patients data we are not able to recognize the flow between the central and peripheral compartments, so it is reasonable to skip the division onto the peripheral and central compartments and consider only one compartment. Eventually, we approximate the amount of the drug in the following way (again, for details see A Appendix in S1 Appendix):

$$\begin{array}{c} L\left(t\right)=\frac{\alpha {\text{e}}^{-k_{1}t}}{d_{L}}(k_{2}+\frac{k_{1}^{n+1}t^{n}}{n!}). \end{array}$$ (5)

**Fig 4:**
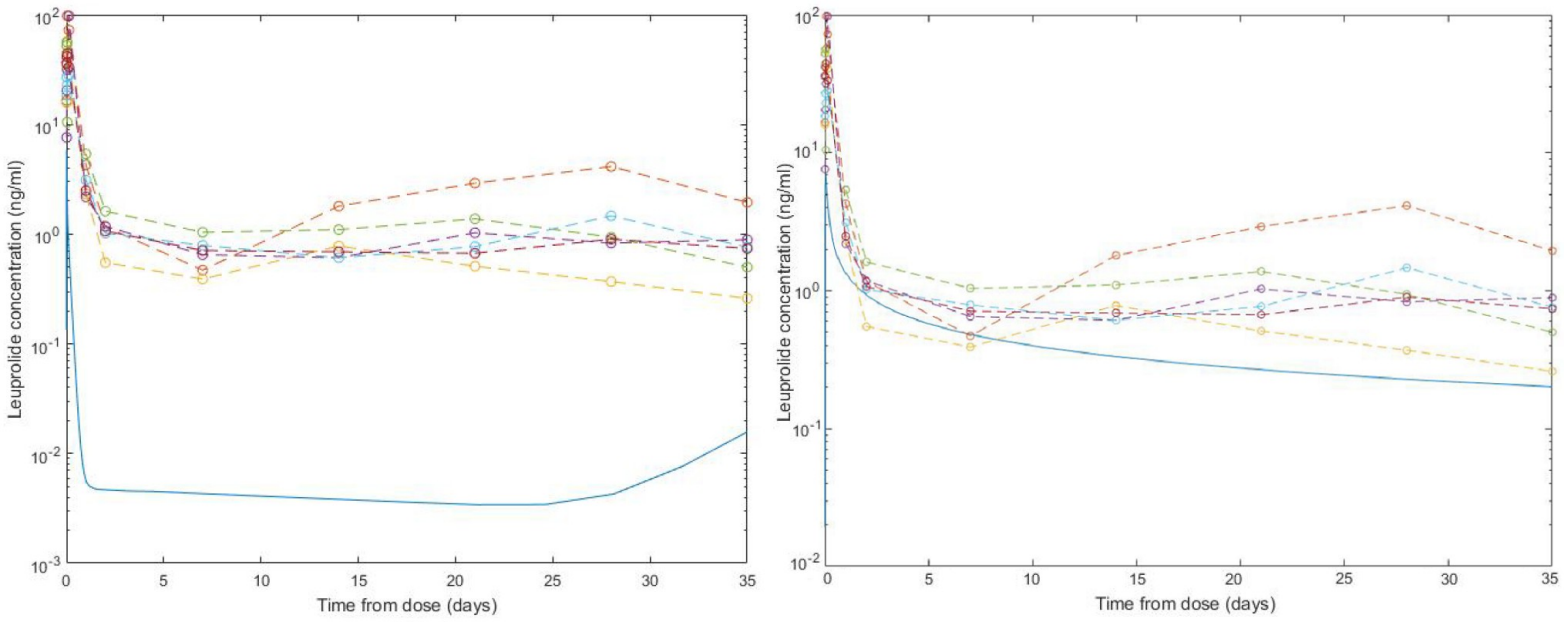
Comparison between the fits obtained for the drug concentration proposed on the basis of mass action law (left) and diffusion-based model (right). Various colors reflect mean population levels for several different Lupron depots available from FDA label.

Unfortunatelly, we were not able to retrieve the results from [23] and the fit to publicly available FDA data on Lupron [24] is not good (cf. Fig 4 left), and therefore we decided to turn to another approach.

#### Diffusion-based PK model

The actual mechanism of the drug release is based on polymer micro-spheres filled with the drug. We therefore consider diffusion of the drug out of the spheres,

$$\begin{array}{c} \frac{\partial \rho }{\partial t}=D\nabla^{2}\rho , \end{array}$$

where *ρ* is the drug concentration and *D* is the diffusion coefficient. Assuming spherical symmetry, the diffusion equation simplifies to

$$\begin{array}{c} \frac{\partial \rho }{\partial t}=D\frac{1}{r^{2}}\frac{\partial }{\partial r}(r^{2}\frac{\partial \rho }{\partial r}). \end{array}$$

As initial condition for this equation we take uniform distribution of the drug within the sphere, that is *ρ*(*r* < *R*, *t* = 0) = *ρ*_0_, where *R* is the radius of the sphere. We also have to specify boundary condition: we assume the diffusion outside the sphere is much faster than within it, so the density outside is always homogeneous, and the boundary condition reads *ρ*(*r* = *R*, *t*) = *ρ*_out_(*t*). To further simplify the equation, we assume *ρ*_out_(*t*) ≪ *ρ*_0_ for all *t*, and solve the equation with the boundary condition *ρ*(*r* = *R*, *t*) = 0. Now the equation can be solved using a series expansion,

$$\begin{array}{c} \rho (r<R,t)=\sum_{n=1}^{\infty }\frac{2\rho_{0}R}{n\pi r}{\left(-1\right)}^{n+1}\;\text{sin}\;(\frac{n\pi r}{R}){\text{e}}^{-\frac{n^{2}\pi^{2}D}{R^{2}}t}. \end{array}$$

The concentration can then be integrated to obtain the amount *M*_in_ of the drug within the sphere

$$\begin{array}{c} M_{\text{in}}(t)=4\pi \int_{0}^{R}\rho (r,t)r^{2}dr=\sum_{n=1}^{\infty }\frac{8\rho_{0}R^{3}}{n^{2}\pi }{\text{e}}^{-\frac{n^{2}\pi^{2}D}{R^{2}}t}. \end{array}$$

Once the drug leaves the micro-sphere, it is cleared from the body at rate *d*_*L*_, therefore

$$\begin{array}{c} {\dot{M}}_{\text{out}}=-{\dot{M}}_{\text{in}}-d_{L}M_{\text{out}}, \end{array}$$

where *M*_out_ is the amount of the drug outside the sphere. Let us denote $\psi (x)=\sum_{n=1}^{\infty }e^{-\pi^{2}n^{2}x}$ and $M_{0}=\frac{4}{3}\pi R^{3}\rho_{0}$, that is the initial mass of the drug within the sphere, to obtain

$$\begin{array}{c} \dot{M_{\text{out}}}=\frac{6D}{R^{2}}M_{0}\psi (\frac{D}{R^{2}}t)-d_{L}M_{\text{out}}, \end{array}$$

leading to final equation for leuprolide within the patients body

$$\begin{array}{c} \dot{L}=k\psi (\frac{\alpha t}{M_{0}})-d_{L}L. \end{array}$$

Note that this approximation leads to much better fit to the data, as can be seen in Fig 4.

#### PD of the drug

In the description of PD for leuprolide the authors of [23] focused on the competition between LHRH and the drug for receptors. If *AGN* denotes the concentration of LHRH normalized by its receptor equilibrium dissociation constant and *BNG* is the concentration of leuprolide normalized in the same way, then $FRAC=\frac{AGN+BGN}{1+AGN+BGN}$ reflects the fraction of activated receptors during the treatment and the equation describing the changes in total number of receptors (*RT*) proposed in [23] has the similar form as those describing LHRH in Systems (8) and (10), but the function *h*_1_ depends here on the difference between *FRAC* and the number of such receptors at baseline. Moreover, it is assumed that a Hill coefficient may be different than 1 (assumed by us for LHRH). As a consequence, the testosterone production depends on the number of activated receptors, that is *FRAC*⋅*RT*. It should be marked that we are not able to control the number of receptors, both activated or inactive. Hence, we decided to include this idea indirectly into our model, taking into account a kind of mathematical description of the competition between LHRH and the drug.

### Modeling testosteron secretion

Mathematical modeling of testosteron secretion started around the eighties of the twentieth century (cf. the review on this topic presented in [25]). The main aim of this research was related to observed oscillations in this system, where three hormones interplay via feedback loop. Testosteron (TES) secretion is regulated directly by luteinizing hormone (LH), which is regulated by luteinizing hormone release hormone (LHRH), while the secretion of the last hormone is regulated by TES. Simple three variable model reflecting this feedback was proposed by Smith [26]. This is a model with general functions describing secretion and degradation of the hormones. The model reads

$$\begin{array}{c} \begin{array}{cc} \dot{x} & =p_{1}\left(z\right)-d_{1}\left(x\right),\\ \dot{y} & =p_{2}\left(x\right)-d_{2}\left(y\right),\\ \dot{z} & =p_{3}\left(y\right)-d_{3}\left(z\right), \end{array} \end{array}$$ (6)

where *x*(*t*), *y*(*t*), *z*(*t*) denote the level of LHRH, LH and TES, respectively, *p*_*i*_ and *d*_*i*_ are their production and degradation functions. All the functions are positive and monotonic, *p*_1_ is decreasing and the others are increasing. Although Smith was able to find oscillatory behavior in this model, but Cartwright and Husain [27] pointed out that this type of oscillations do not capture the real pulse in this system. Later models include time delay/delays [15, 25, 27, 28], due to the well-known property of delayed differential equations—namely, introducing time delay in a proper way we are able to obtain oscillatory solutions. One of the simplest models of that type was studied by Murray [15]. Clearly, Murray analyzed influence of time delay in the following system:

$$\begin{array}{c} \begin{array}{cc} \dot{x} & =p_{1}\left(z\right)-d_{1}x,\\ \dot{y} & =p_{2}x-d_{2}y,\\ \dot{z} & =p_{3}y\left(t-\tau \right)-d_{3}z, \end{array} \end{array}$$ (7)

where now *p*_2_, *p*_3_ and *d*_*i*_ are positive constants and *τ* is the delay of TES secretion. He showed that oscillations via a Hopf bifurcation with *τ* being a bifurcation parameter could appear in this system.

It should be marked however, that we are not interested in short-time oscillatory dynamics in TES levels. Clearly, from the point of view of the ADT treatment mean levels of TES observed in longer time horizon play a crucial role. Hence, we decided not to include time delays and our model is based on Eq (7) but with *τ* = 0. More precisely, we consider

$$\begin{array}{c} \begin{array}{cc} \dot{x} & =h_{1}\left(z\right)-d_{1}x,\;\\ \dot{y} & =p_{2}x-d_{2}y,\\ \dot{z} & =p_{3}y-d_{3}z, \end{array} \end{array}$$ (8)

where:

- *h*_1_ is a smooth positive decreasing function, and we focus on
  $$\begin{array}{c} h_{1}\left(z\right)=\frac{p_{1}}{1+b_{1}z}; \end{array}$$ (9)
- *p*_*i*_ are production rates;
- *d*_*i*_ are clearance rates.

In System (8) with *h*_1_ described by (9) we expect that solutions will stabilize on some positive level in time.

First, we show that for any decreasing *h*_1_, System (8) has exactly one steady state (SS), which is positive. Clearly, let $\left(\bar{x},\bar{y},\bar{z}\right)$ be a steady state. Then

$$\begin{array}{c} \bar{y}=\frac{d_{3}}{p_{3}}\bar{z};\;\bar{x}=\frac{d_{2}}{p_{2}}\bar{y}=\frac{d_{2}d_{3}}{p_{2}p_{3}}\bar{z};\;h_{1}\left(\bar{z}\right)=d_{1}\bar{x}=\frac{d_{1}d_{2}d_{3}}{p_{2}p_{3}}\bar{z}. \end{array}$$

Looking at the relation

$$\begin{array}{c} h_{1}\left(z\right)=\frac{d_{1}d_{2}d_{3}}{p_{2}p_{3}}z \end{array}$$

we see that the left-hand side is decreasing from *h*_1_(0) > 0 to some limit lim *h*_1_(*z*) (which should be 0 in our case but it does not matter as regards the number of SS), while the right-hand side increases from 0 to ∞, so the two curves cross each other exactly once at our SS.

**Proposition 1**. *Unique positive steady state of System* (8) *with h*_1_ *described by* (9) *is locally asymptotically stable regardless of the parameter values*.

The proof of this proposition and the discussion on possible instability are presented in B Appendix in S1 Appendix.

We also expect global stability in the case of Eq (8). It is easy to show that the system is dissipative. Clearly, let $U\left(x,y,z\right)=x+\frac{d_{1}}{2p_{2}}y+\frac{d_{1}d_{2}}{2p_{2}p_{3}}z$. Then

$$\begin{array}{c} \begin{array}{cc} \dot{U}\left(x,y,z\right)= & \;h_{1}\left(z\right)-d_{1}x+\frac{d_{1}}{2p_{2}}(p_{2}x-d_{2}y)+\frac{d_{1}d_{2}}{4p_{2}p_{3}}(p_{3}y-d_{3}z)\\ = & \;h_{1}\left(z\right)-\frac{d_{1}}{2}x-\frac{d_{1}d_{2}}{4p_{2}}y-\frac{d}{4p_{2}p_{3}}z. \end{array} \end{array}$$

Taking $0<\alpha <\frac{\text{min}\{d_{1},d_{2},d_{3}\}}{2}$ we obtain

$$\begin{array}{c} \dot{U}\left(x,y,z\right)\leq h_{1}\left(0\right)-\alpha U\left(x,y,z\right). \end{array}$$

This way we obtain the following conclusion.

**Corollary 2**. *System* (8) *has a compact global attractor*.

Although we are not able to completely precisely argue that the locally stable SS is globally stable, i.e. it forms this global attractor, but from analytical point of view System (8) is almost linear, with only non-linear part described by the function *h*_1_ that depend monotonically on only one variable, and moreover thorough numerical analysis (results not shown) confirms the expected behavior. Hence, it is reasonable to consider a simplified system, in which only two hormones are taken into account, namely

$$\begin{array}{c} \begin{array}{cc} \dot{x} & =h_{1}\left(z\right)-d_{1}x,\\ \dot{z} & =p_{3}x-d_{3}z, \end{array} \end{array}$$ (10)

where *p*_3_ is left for simplicity, although it is not the same coefficient as before. We can think of Eq (10) as of a quasi-steady approximation of Eq (8). In such a case we have *p*_2_*x* = *d*_2_*y*, while the equation for the variable *z* changes to $\dot{z}=\frac{p_{2}p_{3}}{d_{2}}x-d_{3}z$, and this leads to the same steady states of Eqs (8) and (10). However, in the following we will not refer to Eq (8), and therefore we decided to use simple parameter *p*_3_ instead of $\frac{p_{2}p_{3}}{d_{2}}$.

For System 10 we can prove that the only steady state existing for any decreasing *h*_1_ is globally stable.

**Proposition 3**. *System* (10) *has exactly one steady state which is positive and globally stable in* ${\left(R^{+}\right)}^{2}$.

The proof of this proposition is presented in B Appendix in S1 Appendix.

**Fig 5:**
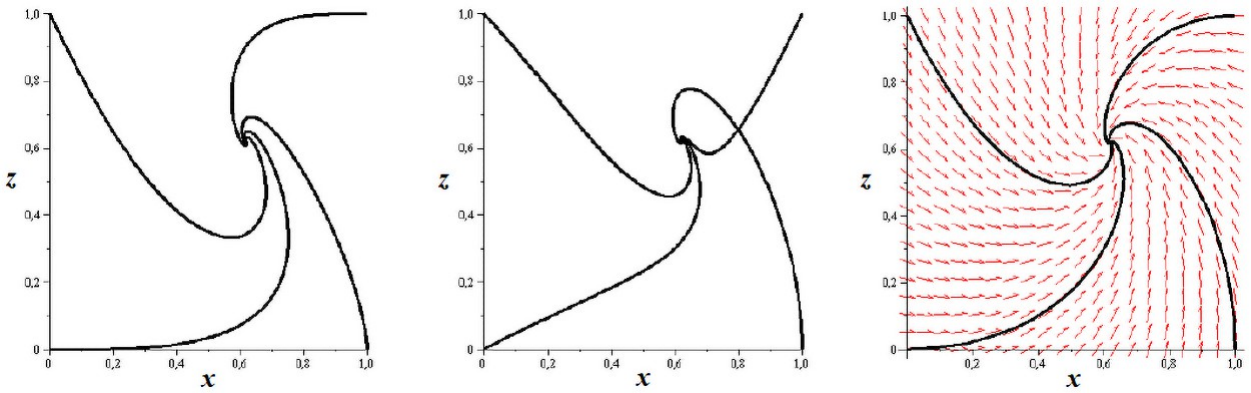
Comparison of the dynamics of the full System (8) and simplified System (10) for all the model parameters equal to 1: projection of the phase space portrait for (8) with initial values for *y*: (left) taken as 0 except for one case where *x*(0) = *y*(0) = *z*(0) = 1; (middle) vary from 0 through 0.5 to 1; phase space portrait for (10) (right). Note that the cross-section of trajectories visible in middle graph is a result of the projection of $R^{3}$ into $R^{2}$.

At the end of this paragraph we present a comparison between the dynamics of both systems considered above. In Fig 5 we see that the dynamics of the full System (8) and simplified System (10) are very similar, not only qualitatively but also quantitatively.

### The model without resistance

In this subsection we combine proposed sub-models describing the underlying tumor growth, the concentration of leuprolide and the secretion of testosteron into one model. First, we need to include the influence of leuprolide to the subsystem describing testosteron. As we mentioned above, pharmacodynamics of this drug could be reflected as some kind of competition between LHRH and the drug. We therefore propose to make a small change in System (10)—instead of linear production term *p*_3_*x* we take

$$\begin{array}{c} h_{3}\left(x,L\right)=\frac{p_{3}x}{1+b_{3}\left(x+L\right)}. \end{array}$$ (11)

Obviously, we can propose more general form of this function, which should be increasing with respect to *x* and decreasing with respect to *x* + *L*. Hence, the testosteron secretion with the influence of leuprolide is described by the following system:

$$\begin{array}{c} \begin{array}{cc} \dot{x} & =h_{1}\left(z\right)-d_{1}x,\\ \dot{z} & =h_{3}\left(x,L\right)-d_{3}z. \end{array} \end{array}$$ (12)

The dynamics of System (12) is crucial for further analysis of the model. Therefore, we present most important result below, while additional information and comparision to the dynamics of System (10) are presented in C Appendix in S1 Appendix, where we show that the dynamics of System (12) for *L* = 0 is similar to the dynamics of System (10), not only qualitatively, but also quantitatively. Clearly, in the same way as for System (10) we are able to prove that for constant *L* ≥ 0 there exists exactly one positive steady state which is globally stable in ${\left(R^{+}\right)}^{2}$.

**Proposition 4**. *For any L* ≥ 0, *System* (12) *has unique positive steady state* $\left({\bar{x}}_{L},{\bar{z}}_{L}\right)$ *which is globally stable in* ${\left(R^{+}\right)}^{2}$.

The proof is presented in C Appendix in S1 Appendix.

Now, combining Eq (1) with Eq (12) we obtain

$$\begin{array}{c} \begin{array}{cc} \dot{P} & =Pf\left(P\right)+d_{P}\left(z-{\bar{z}}_{0}\right)P,\\ \dot{x} & =h_{1}\left(z\right)-d_{1}x,\\ \dot{z} & =h_{3}\left(x,L\right)-d_{3}z, \end{array} \end{array}$$ (13)

where ${\bar{z}}_{0}$ is the testosteron steady state without the treatment, while general functions *f*, *h*_1_ and *h*_3_ satisfy:

1. *f* is of class **C**^1^ and has positive values on some interval (0, *K*), where either *K* < ∞ and then it reflects maximal tumor size or *K* = ∞ and then tumor growth is unbounded;
2. *h*_1_ and *h*_3_ are positive bounded functions of class **C**^1^;
3. $\frac{dh_{1}}{dz}<0$, $\frac{\partial h_{3}}{\partial x}>0$, $\frac{\partial h_{3}}{\partial (x+L)}<0$.

In more specific (e.g. numerical) investigations we used the function *f* described by Eq (3), the functions *h*_*i*_, *i* = 1, 3 described by Eqs (9) and (11), respectively, while the amount of the drug *L* is described by (5).

### The model with resistance

The last step in the model development is to include resistance to System (13). In patients that are treated with LHRH agonists/antagonists for a continuous period, resistance to the drug develops by various biological mechanisms, thereby bringing on the stage of hormone resistance (or castration resistance) in these patients. Multiple mechanisms of resistance include androgen receptor (AR) amplification and hypersensitivity, AR mutations, mutations in coactivators/corepressors, androgen-independent AR activation, and intratumoral and alternative androgen production. Indeed, the onset of this resistance is embodied in the regained PSA production seen in these patients after a certain period (which is very variable, and can span from 3 months after ADT onset, to 3 years after ADT onset). Without modeling this resistance effect (even in the most simplified way as we have done in our model) it is impossible to capture the PSA profiles in these patients over the mentioned periods of time.

In the model we distinguish two different mechanisms: resistance to the drug, that causes testosterone to rise in the presence of ADT, and emerging independence from testosterone, which causes PSA to rise even though the testosterone is low. In general, modeling drug resistance for tumor growth, we distinguish between drug-sensitive and drug-resistant tumor cells. However, in our case we do not describe tumor cells directly, and therefore another approach is necessary. As mentioned above, including resistance we directly follow the ideas from [11]. This means that we have two additional variables *r*_*i*_, *i* = 1, 2, reflecting the strength of resistance. We would like to propose as simple as possible form of equations reflecting that each *r*_*i*_ is increasing with increasing *L*. The simplest form of such equation is linear. Moreover, resistance is acquired slowly and gradually, over a period of 3–18 months (on average) and is patient specific. Taking into account limiting factors for the variables *r*_*i*_ turned out to be crucial to obtain a good fit to the data in the original article [11]. Therefore, we propose the following equation

$$\begin{array}{c} {\dot{r}}_{i}=\beta_{i}L(1-\frac{r_{i}}{l_{i}}), \end{array}$$ (14)

where *β*_*i*_ and *l*_*i*_ reflect coefficients of proportionality and limitation contstants on resistance, respectively. As we describe acquired drug resistance, we assume *r*_*i*_(0) = 0, as at the beginning of the treatment there is no acquired resistance. This implies that both variables *r*_*i*_ are positive and bounded from above by *l*_*i*_ (cf. D Appendix in S1 Appendix for detailed analysis of Eq (14)). The first variable *r*_1_ influences the level of PSA and therefore is included into the first equation of (13), while the second one influences the level of TES due to the presence of the drug, so we include it into the third equation of System (13). Although in [23] specific forms of these influence were proposed, we can include more general influence functions *g*_1_ and *g*_2_ with specific properties. Hence, following the ideas from [23] we obtain the full model with resistance that reads

$$\begin{array}{c} \begin{array}{cc} \dot{P} & =Pf\left(P\right)+d_{P}(z-{\bar{z}}_{0}+g_{1}\left(r_{1}\right))P,\\ \dot{x} & =h_{1}\left(z\right)-d_{1}x,\\ \dot{z} & =h_{3}(x,g_{2}\left(r_{2}\right))-d_{3}z,\\ {\dot{r}}_{1} & =\beta_{1}L(1-\frac{r_{1}}{l_{1}}),\\ {\dot{r}}_{2} & =\beta_{2}L(1-\frac{r_{2}}{l_{2}}), \end{array} \end{array}$$ (15)

with *f*, *h*_1_ and *h*_3_ as defined before and *g*_*i*_, *i* = 1, 2, are smooth non-negative functions having the following properties:

- *g*_1_(0) = 0 and *g*_1_ is increasing; we focus on *g*_1_(*x*) = *a*_1_*x*;
- *g*_2_(0) = *L* and *g*_2_ is decreasing; we focus on $g_{2}\left(x\right)=L\frac{a_{2}+{\text{e}}^{x}}{\left(a_{2}+1\right){\text{e}}^{x}}$.

Note that although the function *g*_1_ is unbounded, its influence is limited because the values of *r*_1_ are bounded above by its steady state value *l*_1_ (cf. D Appendix in S1 Appendix). Similarly, not all values of the function *g*_2_ may appear in System (15). Clearly, *r*_2_ ∈ [0, *l*_2_) yielding $g_{2}\left(r_{2}\right)\in [L,L\frac{a_{2}+{\text{e}}^{l_{2}}}{\left(a_{2}+1\right){\text{e}}^{l_{2}}})$, while $\underset{x\to \infty }{\text{lim}}g_{2}\left(x\right)=\frac{L}{a_{2}+1}$.

## Results

In this section we focus on the analysis of the model proposed above under the simplified assumption that the level of ADT is constant in the organism, that is *L* = const. Although in reality this assumption is not valid, but it gives a preliminary insight to the model dynamics. We start with the analysis of Eq (13) and then switch to the analysis of the full model Eq (15). In the presented analysis we mainly focus on the specific form of functions describing the right-hand side of Eqs (13) or (15), however some of the model properties are valid for general functions satisfying the properties listed in the previous section.

### Analysis of Eq (13)

Let us first assume that the system is able to overcome resistance mechanisms and therefore the dynamics of HSPC is governed by Eq (13).

Note that the (*x*, *z*)-subsystem is independent of the first variable *P*. Hence, the dynamics of this subsystem is just a dynamics of System (12) and from Proposition 4 we know that for any *L* ≥ 0 there is only one positive steady state SS with coordinates $\left({\bar{x}}_{L},{\bar{z}}_{L}\right)$ which is globally stable in the phase space ${\left(R^{+}\right)}^{2}$, that is for any non-negative initial data $\left(x\left(t\right),z\left(t\right)\right)\to \left({\bar{x}}_{L},{\bar{z}}_{L}\right)$ for *t* → ∞. Moreover, for any *L* > 0 we have ${\bar{z}}_{L}<{\bar{z}}_{0}$. Clearly, coordinates of the SS satisfy the following system of equations:

$$\begin{array}{c} \frac{p_{1}}{1+b_{1}{\bar{z}}_{L}}=d_{1}{\bar{x}}_{L},\;\frac{p_{3}{\bar{x}}_{L}}{1+b_{3}\left({\bar{x}}_{L}+L\right)}=d_{3}z_{L}. \end{array}$$

Hence,

$$\begin{array}{c} \frac{p_{1}}{d_{1}\left(1+b_{1}{\bar{z}}_{L}\right)}={\bar{x}}_{L}\;\Rightarrow \;\frac{p_{1}p_{3}}{d_{1}\left(1+b_{1}{\bar{z}}_{L}\right)\left(1+b_{3}L\right)+p_{1}b_{3}}=d_{3}{\bar{z}}_{L}, \end{array}$$

yielding

$$\begin{array}{c} p_{1}p_{3}=d_{3}{\bar{z}}_{L}(d_{1}\left(1+b_{3}L\right)+b_{1}d_{1}{\bar{z}}_{L}\left(1+b_{3}L\right)+p_{1}b_{3}). \end{array}$$

Eventually we obtain a quadratic equation of the form

$$\begin{array}{c} b_{1}\left(1+b_{3}L\right){\bar{z}}_{L}^{2}+(1+b_{3}L+\frac{p_{1}b_{3}}{d_{1}}){\bar{z}}_{L}-\frac{p_{1}p_{3}}{d_{1}d_{3}}=0, \end{array}$$ (16)

and for any *L* > 0 coefficients next to quadratic and linear terms are greater than the appropriate coefficients for *L* = 0, which means that the positive root ${\bar{z}}_{L}$ of Eq 16 for *L* > 0 is smaller comparing to the case *L* = 0. Moreover, ${\bar{z}}_{L}$ decreases with increasing *L*. Clearly, if we treat ${\bar{z}}_{L}$ as a function of *L*, using the theorem of implicit function we calculate

$$\begin{array}{c} \frac{d}{dL}{\bar{z}}_{L}=-\frac{b_{3}{\bar{z}}_{L}(1+b_{3}{\bar{z}}_{L})}{1+b_{3}L+\frac{p_{1}b_{3}}{d_{1}}+2{\bar{z}}_{L}(1+b_{3}L)}<0. \end{array}$$

**Fig 6:**
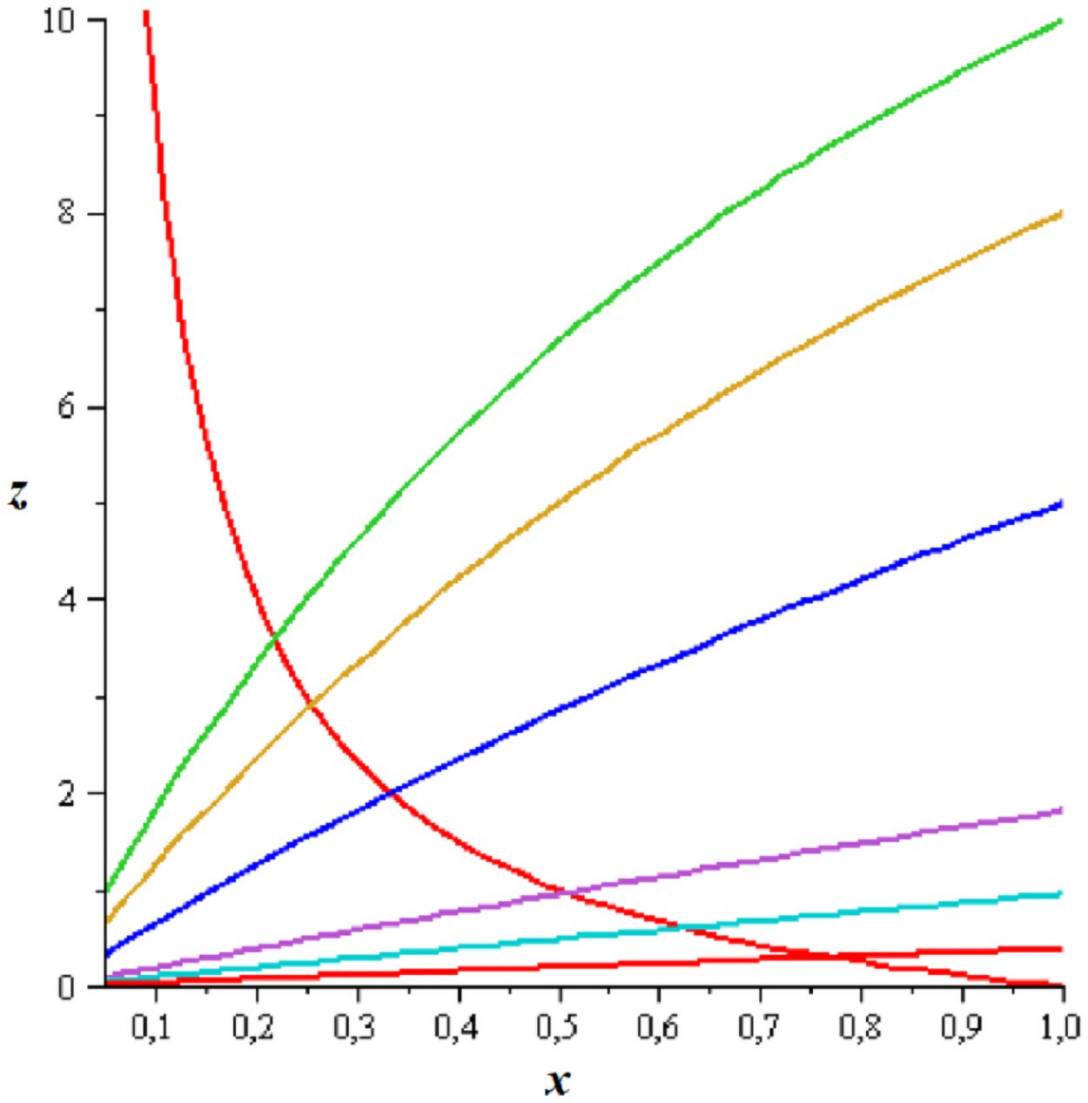
Location of the SS of System (12) in the phase space for parameter values $b_{1}=b_{3}=\frac{p_{1}}{d_{1}}=1$, $\frac{p_{3}}{d_{3}}=20$ and various values of *L*. Red hyperbola represents null-cline for *x*, while other curves represent null-clines for *z* for *L* = 0, 0.5, 2, 9, 19, 49 (from top to bottom).

This implies that ${\bar{z}}_{L}$ has a limit ${\bar{z}}_{\infty }\geq 0$. Calculating this limit we divide Eq 16 by *L* getting

$$\begin{array}{c} b_{1}(b_{3}+\frac{1}{L}){\bar{z}}_{L}^{2}+(b_{3}+\frac{1}{L}+\frac{p_{1}b_{3}}{d_{1}L}){\bar{z}}_{L}-\frac{p_{1}p_{3}}{d_{1}d_{3}L}=0, \end{array}$$

and taking the limit for *L* → ∞ we obtain the relation

$$\begin{array}{c} b_{3}{\bar{z}}_{\infty }(1+b_{1}{\bar{z}}_{\infty })=0, \end{array}$$

which means that ${\bar{z}}_{\infty }=0$. This also implies that ${\bar{x}}_{L}$ is an increasing function of *L* and $\underset{L\to \infty }{\text{lim}}{\bar{x}}_{L}={\bar{x}}_{\infty }=\frac{p_{1}}{d_{1}}$. Dependence of the location of the SS on *L* for exemplary parameter values is presented in Fig 6.

**Fig 7:**
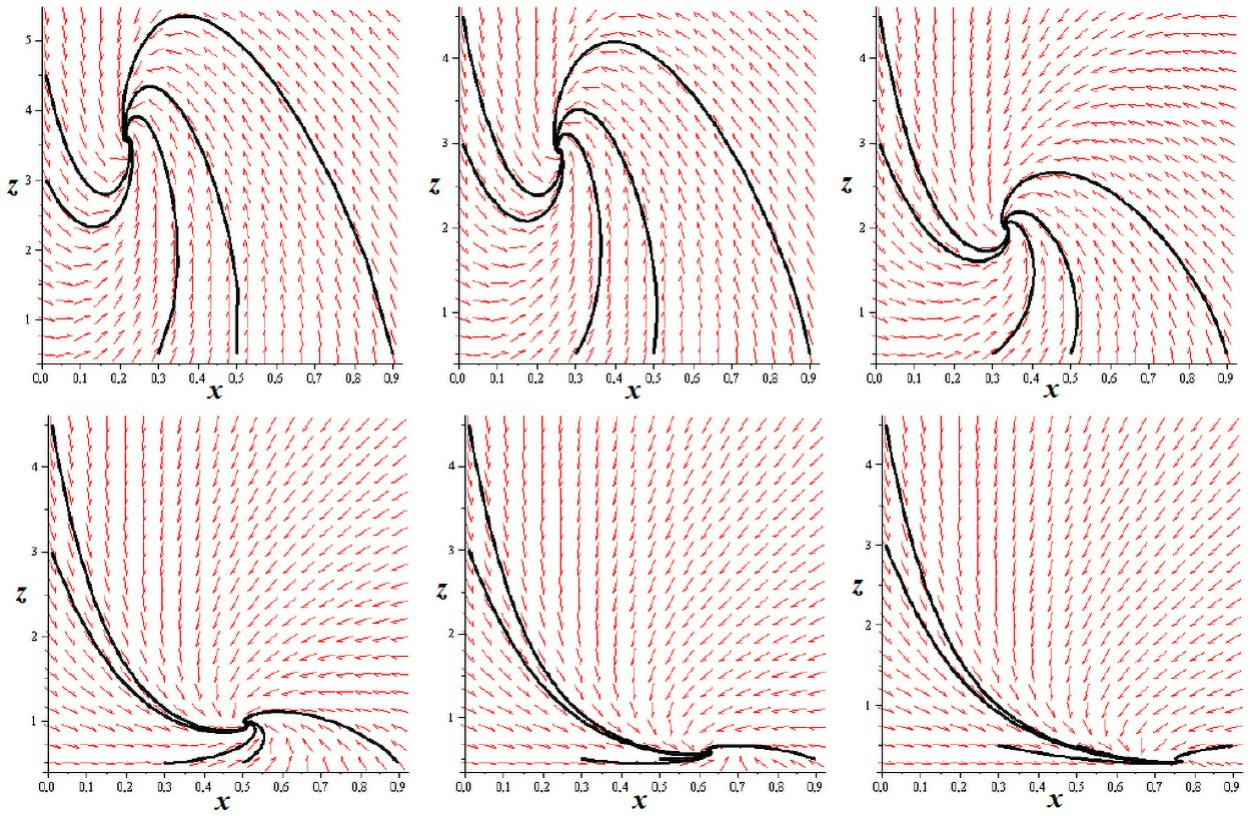
Phase portraits for System (12) with parameters as in Fig 6; *L* = 0 (top left), *L* = 0.5 (top middle), *L* = 2 (top right), *L* = 9 (bottom left), *L* = 19 (bottom middle), *L* = 49 (bottom right).

Qualitatively the phase portrait of System (12) does not change essentially with increasing *L* comparing to *L* = 0; cf. examples in Fig 7.

Knowing the behavior of (*x*, *z*)-subsystem, the asymptotic dynamics of PSA level can be then described by the dynamics of single equation. Below we follow the idea of similar analysis for immunotherapy of prostate cancer presented in [29]. Clearly, the right-hand side of the first equation of System (12) depends only on *P* and *z*. We know that $z\to {\bar{z}}_{L}$ as *t* → ∞. This means that for any *ε* > 0 there exists such *T* that

$$\begin{array}{c} {\bar{z}}_{L}-\varepsilon \leq z\left(t\right)\leq {\bar{z}}_{L}+\varepsilon \;\text{for}\;t>T, \end{array}$$

implying the following bounds for the first equation of System (12):

$$\begin{array}{c} Pf\left(P\right)+d_{P}({\bar{z}}_{L}-\varepsilon -{\bar{z}}_{0})\;P\leq \dot{P}\leq Pf\left(P\right)+d_{P}({\bar{z}}_{L}+\varepsilon -{\bar{z}}_{0})P. \end{array}$$

For any *L* > 0, due to the ineguality ${\bar{z}}_{L}<{\bar{z}}_{0}$ we can find *ε* sufficiently small to have ${\bar{z}}_{L}+\varepsilon -{\bar{z}}_{0}<0$.

Now, instead of the first equation of System (12) we consider an auxiliary equation

$$\begin{array}{c} \dot{P}=P(f\left(P\right)-d_{P}A)≕F\left(P\right), \end{array}$$ (17)

where $A={\bar{z}}_{0}-{\bar{z}}_{L}-\varepsilon >0$ or $A={\bar{z}}_{0}-{\bar{z}}_{L}+\varepsilon >0$. In generic cases Eq (17) has at most two steady states: ${\bar{P}}_{0}=0$ and positive $\bar{P}>P_{R}$ satisfying

$$\begin{array}{c} a{\left(b\;\text{ln}\;\frac{\bar{P}}{P_{R}}+1\right)}^{\gamma }-d_{P}A=0. \end{array}$$

Note that below the threshold of detection, to have $\dot{P}=0$ for *P* ≠ 0 there must be *a* = *d*_*P*_*A*, which is impossible in the generic case. Solving the equation

$$\begin{array}{c} {\left(b\;\text{ln}\;\frac{\bar{P}}{P_{R}}+1\right)}^{\gamma }=\frac{d_{P}}{a}A \end{array}$$

we require $\frac{d_{P}}{a}A>1$, and then

$$\begin{array}{c} \bar{P}=P_{R}\;\text{exp}\;(\frac{{\left(\frac{d_{P}}{a}A\right)}^{1/\gamma }-1}{b}). \end{array}$$

Otherwise, there is only one steady state ${\bar{P}}_{0}=0$.

Looking for stability of the steady states of Eq (17) we obtain the following proposition.

**Proposition 5**. *For* Eq (17),

1. *if a* < *d*_*P*_*A*, *then there exists the positive steady state* $\bar{P}$ *and for initial data satisfying* $P<\bar{P}$ *the trivial state* ${\bar{P}}_{0}$ *attracts the solution, while for initial data satisfying* $P>\bar{P}$ *solutions are repelled from* $\bar{P}$ *and grow to* ∞;
2. *if a* > *d*_*P*_*A*, *then there is no positive steady state and all solutions grow to* ∞.

*Proof*. Assuming that $\bar{P}\neq P_{R}$ we can calculate the derivative of the right-hand side of Eq (17) with respect to *P* obtaining

$$\begin{array}{c} F^{\prime }\left(P\right)=\{\begin{array}{cc} a{\left(b\;\text{ln}\;\frac{P}{P_{R}}+1\right)}^{\gamma }-d_{P}A+ab\gamma {\left(b\;\text{ln}\;\frac{P}{P_{R}}+1\right)}^{\gamma -1} & \text{for}\;P>P_{R},\\ a-d_{P}A & \text{for}\;P<P_{R}. \end{array} \end{array}$$

We see that

$$\begin{array}{c} F^{\prime }\left({\bar{P}}_{0}\right)=a-d_{P}A,\;F^{\prime }\left(\bar{P}\right)=ab\gamma {\left(b\;\text{ln}\;\frac{\bar{P}}{P_{R}}+1\right)}^{\gamma -1}>0. \end{array}$$

This means that stability of ${\bar{P}}_{0}$ depends on the sign of *a* − *d*_*P*_*A*, while $\bar{P}$ is unstable whenever exists. More precisely,

1. if *a* < *d*_*P*_*A*, then ${\bar{P}}_{0}$ is stable, while $\bar{P}$ exists and is unstable,
2. if *a* > *d*_*P*_*A*, then ${\bar{P}}_{0}$ is unstable, while $\bar{P}$ does not exist.

Taking the limit *ε* → 0, we obtain an asymptotic version of the first equation of System (12) that reads

$$\begin{array}{c} \dot{P}=Pf\left(P\right)+d_{P}({\bar{z}}_{L}-{\bar{z}}_{0})P, \end{array}$$ (18)

with

$$\begin{array}{c} \bar{P}=P_{R}\;\text{exp}\;(\frac{{\left(\frac{d_{P}}{a}\left({\bar{z}}_{0}-{\bar{z}}_{L}\right)\right)}^{1/\gamma }-1}{b}), \end{array}$$ (19)

and we can reformulate Proposition 5 for this equation.

**Proposition 6**. *For arbitrary L* ≥ 0, *solutions of* Eq (18) *satisfy*:

- *if* $a<d_{P}\left({\bar{z}}_{0}-{\bar{z}}_{L}\right)$ *then there exists the positive steady state* $\bar{P}$ *and for initial data satisfying* $P<\bar{P}$ *the trivial state* ${\bar{P}}_{0}$ *attracts the solution, while for initial data satisfying* $P>\bar{P}$ *solutions are repelled from* $\bar{P}$ *and grow to* ∞;
- *if* $a>d_{P}\left({\bar{z}}_{0}-{\bar{z}}_{L}\right)$ *then there is no positive steady state and all solutions grow to* ∞.

Note that, according to Formula (19), if the positive steady state $\bar{P}$ exists, then it is increasing in *L* (as ${\bar{z}}_{L}$ is decreasing), which means that the basin of attraction of ${\bar{P}}_{0}$ enlarges. Let us check for which parameter values there is no positive steady state of Eq (18). We need

$$\begin{array}{c} {\bar{z}}_{0}-{\bar{z}}_{L}<\frac{a}{d_{P}} \end{array}$$

that is

$$\begin{array}{c} \frac{\sqrt{{\left(\frac{p_{1}b_{3}}{d_{1}}+1\right)}^{2}+4b_{1}\frac{p_{1}p_{3}}{d_{1}d_{3}}}-\left(\frac{p_{1}b_{3}}{d_{1}}+1\right)}{2b_{1}}-\frac{\sqrt{{\left(\frac{p_{1}b_{3}}{d_{1}}+1+b_{3}L\right)}^{2}+4b_{1}\frac{p_{1}p_{3}}{d_{1}d_{3}}\left(1+b_{3}L\right)}-\left(\frac{p_{1}b_{3}}{d_{1}}+1+b_{3}L\right)}{2b_{1}\left(1+b_{3}L\right)}<\frac{a}{d_{P}}, \end{array}$$

and, as we see, the left-hand side of the inequality above depends neither on *a* nor *d*_*P*_, which means that for sufficiently large *a* or small *d*_*P*_ there is no positive steady state and all solutions grow to ∞.

Let us look at this inequality for changing *L*. The left-hand side as a function of *L* is increasing, as ${\bar{z}}_{0}$ does not depend on *L* while ${\bar{z}}_{L}$ is decreasing. With *L* increasing to ∞ the left-hand side increases to ${\bar{z}}_{0}$ as ${\bar{z}}_{\infty }=0$. Hence, if ${\bar{z}}_{0}<\frac{a}{d_{P}}$, then there is no positive steady state for any *L* ≥ 0. This inequality is equivalent to $\frac{a}{d_{P}}>\tilde{\frac{a}{d_{P}}}$, where

$$\begin{array}{c} \tilde{a}≔d_{P}{\bar{z}}_{0}. \end{array}$$

**Corollary 7**. *For* Eq (18), *depending on L*,

- *if* $a>\tilde{a}$, *then there is no positive steady state and all solutions grow to* ∞;
- *if* $a<\tilde{a}$, *then for sufficiently large L* > 0 *there exists a positive steady state* $\bar{P}$ *and solutions have the following dynamics depending on the initial value P*_0_ > 0:
  *if* $P_{0}<\bar{P}$ *then P decreases to 0*;
  *if* $P_{0}>\bar{P}$ *then P grows to* ∞.

This corollary means that, depending on the early beginning growth rate of tumor described by the parameter *a*, if *a* is large, then there is no possibility to cure the disease even using arbitrary large doses *L*, while if *a* is small, the possibility of cure depends on the initial tumor size, and moreover larger tumors can be cured for larger *L*. On the other hand, we can reformulate this corollary taking into account the value of *d*_*P*_. Clearly, we are interested in HSPC patients, which means that the influence of testosterone (reflected by *d*_*P*_) on the tumor growth and thus PSA level is significant. Hence, we can conclude that in general, in HSPC patients, the more hormone-sensitive the tumor, the more probable it is to be cured in an ideal scenario when there is no drug resistance. Moreover, small values of *d*_*P*_ should reflect CRPC patients who cannot be cured even with very high doses of the drug.

Note that the results presented above does not essentially depend on the form of the tumor per capita growth rate *f*(*P*). In fact, we can follow this line of reasoning for any positive, increasing and unbounded function *f*.

### Analysis of the full model (15)

Now, we turn to the similar analysis for the full system with resistance described by Eq (15). We can divide our study into two cases which could be analyzed separately.

**Case 1**. If only the first resistance mechanism is present, then the (*x*, *z*) subsystem is independent of this resistance, and from the previous subsection we know that these variables tend to their steady state values and the system could be reduced asymptotically to one equation for *P*. As *r*_1_ influences the right-hand side of the first equation of (15) monotonically, this means that one can proceed exactly in the same way as in the previous subsection to obtain the same qualitative result. The only difference is related with the threshold condition for the existence of the positive steady state being also the threshold value for *P*_0_, which is now dependent on the limit of *r*_1_. More precisely, the threshold condition for the existence of $\bar{P}$ changes from $a=\tilde{a}$ to $a+a_{1}d_{p}l_{1}=\tilde{a}$ (where *a*_1_ is the parameter of the linear function *g*_1_ and *l*_1_ is the limit of the variable *r*_1_), while $\bar{P}=P_{R}\;\text{exp}\;(\frac{{\left(\frac{d_{P}}{a}\left({\bar{z}}_{0}-{\bar{z}}_{L}-a_{1}d_{P}l_{1}\right)\right)}^{1/\gamma }-1}{b})$.

**Case 2**. If only the second resistance mechanism is present, then the (*x*, *z*) subsystem is dependent on this resistance. However, when *L* is not large (and this should be the case of real ADT) we do not expect that it will influence qualitative dynamics of this subsystem. Let us focus on the dynamics of the (*x*, *z*, *r*_2_) subsystem which is independent of the other equations of the full System (15), that is we consider

$$\begin{array}{c} \begin{array}{cc} \dot{x} & =\frac{p_{1}}{1+b_{1}z}-d_{1}x,\\ \dot{z} & =\frac{p_{3}x}{1+b_{3}(x+g_{2}\left(r_{2}\right))}-d_{3}z,\\ {\dot{r}}_{2} & =\beta_{3}(1-\frac{r_{2}}{l_{2}}), \end{array} \end{array}$$ (20)

with *β*_3_ = *β*_2_*L* = const. in the invariant subset $\Omega_{r_{2}}=\left[x_{m},x_{M}\right]\times \left[z_{m},z_{M}\right]\times \left[0,l_{2}\right]\subset {\left(R_{+}\right)}^{3}$ (cf. B Appendix and D Appendix in S1 Appendix).

First, we show that there exists a unique positive steady state of Eq (20) which is locally stable. As in $\Omega_{r_{2}}$ the resistance *r*_2_ influences the (*x*, *z*) subsystem only to some extent we also expect global stability. However, as the dependence of Eq (20) on *r*_2_ is highly non-linear, it is difficult to find a suitable Lyapunov function for this system for any parameters values. We will prove global stability for sufficiently small values of *L*.

**Proposition 8**. *System* (20) *has exactly one positive steady state which is locally stable independently of the model parameters*.

*Proof*. For a steady state $\left(\bar{x},\bar{z},{\bar{r}}_{2}\right)$ we have

$$\begin{array}{c} \bar{x}=\frac{p_{1}}{d_{1}\left(1+b_{1}\bar{z}\right)},\;\bar{z}=\frac{p_{3}\bar{x}}{d_{3}(1+b_{3}(\bar{x}+g_{2}\left({\bar{r}}_{2}\right)))},\;{\bar{r}}_{2}=l_{2}. \end{array}$$

Let us denote $q_{i}=\frac{p_{i}}{b_{i}d_{i}}$, $c_{i}=\frac{1}{b_{i}}$, *i* = 1, 2. This yields $\bar{x}=\frac{q_{1}}{c_{1}+\bar{z}}$, $\bar{z}=\frac{q_{3}\bar{x}}{c_{3}+\bar{x}+g_{2}\left({\bar{r}}_{2}\right)}$, and therefore

$$\begin{array}{c} \bar{z}=\frac{q_{1}q_{3}}{q_{1}+\left(c_{1}+\bar{z}\right)\left(c_{3}+g_{2}\left(l_{2}\right)\right)}\;\Rightarrow \;(c_{3}+g_{2}\left(l_{2}\right)){\bar{z}}^{2}+(q_{1}+c_{1}\left(c_{3}+g_{2}\left(l_{2}\right)\right))\bar{z}-q_{1}q_{3}=0, \end{array}$$

and there exists exactly one positive solution $\bar{z}$ of this quadratic equation which depends on *L* via the values of *g*_2_(*l*_2_).

Calculating Jacobian matrix of Eq (20) we obtain

$$\begin{array}{c} J\left(\bar{x},\bar{y},{\bar{r}}_{2}\right)=(\begin{array}{ccc} -d_{1} & -\frac{b_{1}p_{1}}{{\left(1+b_{1}\bar{z}\right)}^{2}} & 0\\ \frac{p_{3}\left(1+b_{3}g_{2}\left({\bar{r}}_{2}\right)\right)}{{\left(1+b_{3}\left(\bar{x}+g_{2}\left(r_{2}\right)\right)\right)}^{2}} & -d_{3} & ⋆\\ 0 & 0 & -\frac{\beta_{3}}{l_{2}} \end{array}), \end{array}$$

where ⋆ is some expression depending on the model parameters and not influencing stability of the steady state. Clearly, the steady state is locally stable as the third eigenvalue $\lambda_{3}=-\frac{\beta_{3}}{l_{2}}<0$, while for the (*x*, *z*)-subsystem we have the trace −(*d*_1_ + *d*_3_) < 0 and the determinant $d_{1}d_{3}+\frac{b_{1}\left(1+b_{3}g_{2}\left({\bar{r}}_{2}\right)\right)p_{1}p_{3}}{{\left(1+b_{1}\bar{z}\right)}^{2}{\left(1+b_{3}\left(\bar{x}+g_{2}\left(r_{2}\right)\right)\right)}^{2}}>0$.

**Theorem 9**. *For sufficiently small values of L the steady state* $\left(\bar{x},\bar{z},{\bar{l}}_{2}\right)$ *is globally stable*.

*Proof*. Let us denote $u=x-\bar{x}$, $v=z-\bar{z}$, $w=r_{2}-{\bar{r}}_{2}$ and rewrite Eq (20) in the new variables:

$$\begin{array}{c} \begin{array}{cc} \dot{u} & =-d_{1}u-\frac{q_{1}v}{\left(c_{1}+\bar{z}\right)\left(c_{1}+\bar{z}+v\right)},\\ \dot{v} & =-d_{3}v+\frac{q_{3}({\hat{c}}_{3}u-\bar{x}(g_{2}\left(w+{\bar{r}}_{2}\right)-g_{2}\left({\bar{r}}_{2}\right)))}{\left({\hat{c}}_{3}+\bar{x}\right)({\hat{c}}_{3}+\bar{x}+u+g_{2}\left(w+{\bar{r}}_{2}\right)-g_{2}\left({\bar{r}}_{2}\right))},\\ \dot{w} & =-\frac{\beta_{3}}{l_{2}}w, \end{array} \end{array}$$ (21)

where ${\hat{c}}_{3}=c_{3}+g_{2}\left({\bar{r}}_{2}\right)$ and now $q_{i}=\frac{p_{i}}{b_{i}}$, $c_{i}=\frac{1}{b_{i}}$, *i* = 1, 2.

We propose the following Lyapunov function for this system:

$$\begin{array}{c} L\left(u,v,w\right)=\frac{q_{3}{\hat{c}}_{3}}{{\hat{c}}_{3}+\bar{x}}(u-\left({\hat{c}}_{3}+\bar{x}\right)\;\text{ln}\;\frac{{\hat{c}}_{3}+\bar{x}+u}{{\hat{c}}_{3}+\bar{x}})+q_{1}(v-\left(c_{1}+\bar{z}\right)\;\text{ln}\;\frac{c_{1}+\bar{z}+v}{c_{1}+\bar{z}})+\frac{B}{2}w^{2}, \end{array}$$

where *B* = const. > 0 is to be chosen appropriately.

We easily see that **L**(*u*, *v*, *w*) ≥ 0 whenever **L** is defined, in particular in the invariant set. Let us calculate the derivative of **L** along solutions of (21). We obtain

$$\begin{array}{c} \begin{array}{cc} \dot{L}\left(x,z,r_{2}\right)= & \;\frac{q_{3}{\hat{c}}_{3}u}{\left({\hat{c}}_{3}+\bar{x}\right)\left({\hat{c}}_{3}+\bar{x}+u\right)}(-d_{1}u-\frac{q_{1}v}{\left(c_{1}+\bar{z}\right)\left(c_{1}+\bar{z}+v\right)})\\ & \;+\frac{q_{1}v}{\left(c_{1}+\bar{z}\right)\left(c_{1}+\bar{z}+v\right)}(-d_{3}v+\frac{q_{3}({\hat{c}}_{3}u-\bar{x}(g_{2}\left(w+{\bar{r}}_{2}\right)-g_{2}\left({\bar{r}}_{2}\right)))}{\left({\hat{c}}_{3}+\bar{x}\right)({\hat{c}}_{3}+\bar{x}+u+g_{2}\left(w+{\bar{r}}_{2}\right)-g_{2}\left({\bar{r}}_{2}\right))})\\ & \;-B\beta_{3}w^{2}\\ = & \;-\frac{q_{3}{\hat{c}}_{3}d_{1}u^{2}}{\left({\hat{c}}_{3}+\bar{x}\right)\left({\hat{c}}_{3}+\bar{x}+u\right)}-\frac{q_{1}d_{3}v^{2}}{\left(c_{1}+\bar{z}\right)\left(c_{1}+\bar{z}+v\right)}-B\beta_{3}w^{2}\\ & \;-\frac{q_{1}q_{3}{\hat{c}}_{3}uv}{\left({\hat{c}}_{3}+\bar{x}\right)\left({\hat{c}}_{3}+\bar{x}+u\right)\left(c_{1}+\bar{z}\right)\left(c_{1}+\bar{z}+v\right)}\\ & \;+\frac{q_{1}q_{3}({\hat{c}}_{3}uv-\bar{x}v(g_{2}\left(w+{\bar{r}}_{2}\right)-g_{2}\left({\bar{r}}_{2}\right)))}{\left(c_{1}+\bar{z}\right)\left(c_{1}+\bar{z}+v\right)\left({\hat{c}}_{3}+\bar{x}\right)({\hat{c}}_{3}+\bar{x}+u+g_{2}\left(w+{\bar{r}}_{2}\right)-g_{2}\left({\bar{r}}_{2}\right))}. \end{array} \end{array}$$

Note that $g_{2}\left(w+{\bar{r}}_{2}\right)-g_{2}\left({\bar{r}}_{2}\right)=g_{2}^{\prime }\left(\zeta \right)w$ (where *ζ* is an intermediate value), so we can treat $\text{L}(\text{u},\dot{\text{v}},\text{w})$ as a quadratic form of (*u*, *v*, *w*) with variable coefficients. We can check positivity of $-\dot{L}$ studying the matrix

$$\begin{array}{c} (\begin{array}{ccc} \frac{{\hat{c}}_{3}d_{1}C_{x}}{{\hat{c}}_{3}+x} & \frac{{\hat{c}}_{3}C_{x}C_{z}g_{2}^{\prime }\left(\zeta \right)w}{2\left(c_{1}+z\right)\left({\hat{c}}_{3}+x\right)\left({\hat{c}}_{3}+x+g_{2}^{\prime }\left(\zeta \right)w\right)} & 0\\ \frac{{\hat{c}}_{3}C_{x}C_{z}g_{2}^{\prime }\left(\zeta \right)w}{2\left(c_{1}+z\right)\left({\hat{c}}_{3}+x\right)\left({\hat{c}}_{3}+x+g_{2}^{\prime }\left(\zeta \right)w\right)} & \frac{d_{3}C_{z}}{c_{1}+z} & \frac{\bar{x}C_{x}C_{z}g_{2}^{\prime }\left(\zeta \right)}{2\left(c_{1}+z\right)\left({\hat{c}}_{3}+x+g_{2}^{\prime }\left(\zeta \right)w\right)}\\ 0 & \frac{\bar{x}C_{x}C_{z}g_{2}^{\prime }\left(\zeta \right)}{2\left(c_{1}+z\right)\left({\hat{c}}_{3}+x+g_{2}^{\prime }\left(\zeta \right)w\right)} & B\beta_{3} \end{array}), \end{array}$$

where $C_{x}=\frac{q_{3}}{{\hat{c}}_{3}+\bar{x}}$, $C_{z}=\frac{q_{1}}{c_{1}+\bar{z}}$. Now, we need to check main minors of this matrix:

1. $\Delta_{1}=\frac{q_{3}{\hat{c}}_{3}d_{1}}{\left({\hat{c}}_{3}+\bar{x}\right)\left({\hat{c}}_{3}+x\right)}$,
2. $\Delta_{2}=\frac{q_{1}q_{3}{\hat{c}}_{3}d_{1}d_{3}}{\left(c_{1}+\bar{z}\right)\left(c_{1}+z\right)\left({\hat{c}}_{3}+\bar{x}\right)\left({\hat{c}}_{3}+x\right)}-{\left(\frac{q_{1}q_{3}{\hat{c}}_{3}g_{2}^{\prime }\left(\zeta \right)w}{2\left(c_{1}+\bar{z}\right)\left(c_{1}+z\right)\left({\hat{c}}_{3}+\bar{x}\right)\left({\hat{c}}_{3}+x\right)\left({\hat{c}}_{3}+x+g_{2}^{\prime }\left(\zeta \right)w\right)}\right)}^{2}$,
3. $\Delta_{3}=B\beta_{3}\Delta_{2}-{\left(\frac{q_{1}q_{3}\bar{x}g_{2}^{\prime }\left(\zeta \right)}{2\left(c_{1}+\bar{z}\right)\left(c_{1}+z\right)\left({\hat{c}}_{3}+\bar{x}\right)\left({\hat{c}}_{3}+x+g_{2}^{\prime }\left(\zeta \right)w\right)}\right)}^{2}\frac{q_{3}{\hat{c}}_{3}d_{1}}{\left({\hat{c}}_{3}+\bar{x}\right)\left({\hat{c}}_{3}+x\right)}$.

Analyzing these minors we conclude:

1. The first minor Δ_1_ > 0 for any parameters, and moreover $\Delta_{1}\in [\frac{q_{3}{\hat{c}}_{3}d_{1}}{\left({\hat{c}}_{3}+\bar{x}\right)\left({\hat{c}}_{3}+x_{M}\right)},\frac{q_{3}{\hat{c}}_{3}d_{1}}{\left({\hat{c}}_{3}+\bar{x}\right)\left({\hat{c}}_{3}+x_{n}\right)}]$ in the invariant subset.
2. If *L* = 0, then $g_{2}^{\prime }\left(r_{2}\right)=0$, as this function depends on *L* linearly. Therefore, for *L* = 0 we have Δ_1_ > 0, which implies that for small *L* > 0 there is Δ_2_ > 0 as the dependence on *L* is smooth.
3. Knowing that Δ_2_ is bounded from below by a positive constant, we can chose *B* large enough for Δ_3_ > 0.

This completes the proof.

Note that the second resistance partially counteracts the effect of treatment on testosterone level. The larger the values of parameters *a*_2_ and *l*_2_, the smaller the asymptotic values the function *g*_2_(*r*_2_) approaches. This leads to increase of the asymptotic level of testosterone.

Combining the results of our analysis for both resistance mechanisms separately we conclude that the first resistance may directly lead to the change of the threshold level $\bar{P}$ leading to unbounded growth of PSA for smaller initial tumors, while the second resistance affects PSA through the asymptotic level of testosterone that increase again when this mechanism is present. In reality (e.g. when *L* is small), either there is no positive steady state of Eq (15) and *P* always increases to ∞, or this state does exist and—depending on initial data—either *P* goes to 0 (this is in fact the case not observed by medical doctors as then *P* goes to 0 without the treatment) or *P* goes to ∞.

## Discussion

Now, we would like to discuss scenarios that are possible according to the model presented above, and confront it with real-life/clinical scenarios.

**Case 1. No disease**. When there is no disease, mean levels of hormones remain at their steady state, while the level of PSA should be undetectable, that is we consider it as 0. This case is perfectly reflected by the model without resistance (13) and without the treatment (*L* = 0), where there is always healthy steady state $\left({\bar{P}}_{0},{\bar{x}}_{0},{\bar{z}}_{0}\right)$. Moreover, even if the state deviates from this steady level a little bit, in the case without disease our model has (i.e. the inequality $a<d_{P}{\bar{z}}_{0}$ holds) a positive steady state $\left(\bar{P},{\bar{x}}_{0},{\bar{z}}_{0}\right)$, and therefore for small deviations the level of PSA comes back to 0. The steady state value $\bar{P}$ is a threshold below which such behavior is observed.

**Case 2. Disease is present**. In the disease case, before the treatment, the patient’s state is again governed by Eq (13) with *L* = 0, however the level of testosterone rises and this lead to increasing level of PSA. We assume that the medical intervention is now inevitable, which means that either there is no positive thereshold level $\bar{P}$ of PSA, or the detected level fo PSA exceeds this threshold, and according to the model, we observe unbounded growth of PSA. We consider HSPC patients, and this case could be interpreted as sufficiently large values of the coefficient *d*_*P*_, such that according to Corollary 7 there exists sufficient level of the drug *L* > 0 for which the threshold value of PSA appears and in ideal scenario if there is no acquired drug resistance (ADR), then the disease may be cured. However, ADR always occurs, and then we need to switch to the full model with resistance (15). Note that the analysis of the full model present in Results section does not exclude the possibility of cure even in the case with ADR. However, in reality after some time HSPC transforms into CRPC, and this transformation could be interpreted as decrease of the values of the coefficient *d*_*P*_. Hence, we expect that the positive steady state $\bar{P}$ disappears and the level of PSA becomes uncontrolable, meaning unbounded tumor growth. Note that we added two types of ADR mechanisms, following the original article [11] where the authors were not able to fit the model to the data using only one type of ADR. However, in our case this should be verified, as it seems that the second ADR mechanism (leading to the changes in the testosterone level) is more close to reality. We hope that our model, better grounded in the biology of the described processes, will be able to reflect the data succesfully to be usefull in predicting biochemical failure and proposing better treatment schedules.

## Conclusion

In this paper we have focused on modeling ADT for HSPC patients. We proposed an advanced mathematical model of this treatment, basing on earlier research by Elishmereni et al. [11], where the authors focused on retrieving clinical data from Mayo hospital. In the present paper we reformulated the previous model including more biological and pharmacokinetic informations. We focused on detailed derivation and description of the new model, along with mathematical analysis allowing to predict the model dynamics.

With the advent of new-generation hormonal therapies such as enzalutamide and abiraterone, the arsenal of therapeutic ADT possibilities for HSPC patients is constantly growing (cf. [30]), and this reality requires new models that could assist in clinical decision making. It is worth mentioning that administration of ADT in HSPC patients harbors several obstacles. Hormone-based therapeutics entail a negative impact on the quality of life, and require dealing with life-threatening adverse events (such as cardiovascular events and neurocognitive disfunction; cf. [31]). Moreover, long-term continuous use of ADT for suppressing androgen release has been suggested to carry a risk of early onset of resistance mechanisms to the drug, and earlier progression of the patient to the castrate-resistant stage of the disease (cf. [32]). Therefore, early identification of conversion from hormone-sensitivity to castration resistance is critical, for adequately planning ADT. Our updated model could potentially serve as a platform for simulating and optimizing ADT selection and schedules for the given patient.

Using mixed effects modeling we partially compared the model with clinical data, proposing the underlying tumor growth law on the basis of the relevant part of the data from Mayo hospital. More precisely, we have been able to reflect the underlying tumor growth for a cohort of 19 patients for which the data before ADT onset was available. Then we fit the pharmacokinetic part of the model to the publicly available FDA data for patients under continuous ADT. However, in this paper we mainly focused on mathematical analysis of the proposed model. We first analyzed a simplified model without resistance, showing that, depending on the initial data and the model parameters, there is either a possible cure or the testosterone level increases to infinity. Then we showed that for the full model with two resistance mechanisms the dynamics is qualitatively the same, there are only quantitative differences. Exactly such type of the behavior is observed in HSPC patients, that is after some time of controlled dynamics the level of PSA rises, and although the model predicts the possibility of cure using large doses of the drug, in practice such doses are far too large to be able to safely applied. We conclude that our model is able to reflect real clinical scenarios in which the level of PSA eventually increases, concomitant with failure of ADT. The new personalized model should next undergo sufficient steps to ensure its validation and usefulness in predicting the time to biochemical failure, and moreover, could help in delaying this process and offering better ADT scheduling on an individual basis. The next steps of validation and personalization of the model are within our future plans.

## Supporting information

S1 Appendix (PDF)
