# Supplementary material for: Mathematical model of hormone sensitive prostate cancer treatment using leuprolide: A small step towards personalization
Source: PLoS One. 2022 Feb 15;17(2):e0263648. doi: 10.1371/journal.pone.0263648 (PMC8846544; doi:10.1371/journal.pone.0263648)
Supplement: S1 Appendix — (PDF) [file pone.0263648.s001.pdf]

A Appendix. Here, we present some calculations related to System (4). Assuming single application of the drug at time $t = 0$ we obtain $D(t) = \alpha\,\mathrm{e}^{-k_1 t}$, where α is related to the portion of the drug absorbed via delayed path. Before the drug application, there is no drug in the transit compartments, as well as in the central and peripheral compartments. Hence, we have zero as initial data for these compartments. This implies

$$L_1(t) = L_1(0)\,\mathrm{e}^{-k_1 t} + \mathrm{e}^{-k_1 t}\int_0^t k_1 D(s)\,\mathrm{e}^{k_1 s}\,ds = \alpha k_1 t\,\mathrm{e}^{-k_1 t}.$$

Using the method of mathematical induction we easily show that

$$L_j(t) = \alpha\frac{k_1^j t^j}{j!}\,\mathrm{e}^{-k_1 t}, \quad j = 1, \dots n. \tag{22}$$

Clearly, if $L_j(t)$ is described by (22) then

$$L_{j+1} = \mathrm{e}^{-k_1 t}\int_0^t k_1 L_j(s)\,\mathrm{e}^{k_1 s}\,ds = \alpha k^{j+1}\,\mathrm{e}^{-k_1 t}\int_0^t \frac{s^j}{j!}ds = \alpha\frac{k_1^{j+1}t^{j+1}}{(j+1)!}\,\mathrm{e}^{-k_1 t},$$

which proves that Eq. (22) is valid for all $j \le n$, as we have shown that it is valid for $j = 1$ previously.

Recall, that we assumed $k_3 = 0$, which means that we obtain the following system of linear non-homogeneous equations of the form

$$\begin{aligned}\dot{L}_c &= \alpha\frac{k_1^{n+1}t^n}{n!}\,\mathrm{e}^{-k_1 t} + \alpha k_2\,\mathrm{e}^{-k_1 t} + QL_p - (Q + d_L)L_c,\\ \dot{L}_p &= QL_c - QL_p.\end{aligned}$$

Note that this system could be solved, but as we are not able to recognize the flow between the central and peripheral compartments, we skip this division and consider only one compartment, which yields

$$\dot{L} = \alpha\frac{k_1^{n+1}t^n}{n!}\,\mathrm{e}^{-k_1 t} + \alpha k_2\,\mathrm{e}^{-k_1 t} - d_L L,$$

where L is the amount of the drug in the organizm, and solving this equation we obtain

$$L(t) = \alpha\,\mathrm{e}^{-k_1 t}\left(k_2\tfrac{1-\mathrm{e}^{-(d_L-k_1)t}}{d_L-k_1} + k_1^{n+1}\sum_{i=0}^{n}\tfrac{(-1)^{n-i}t^i}{i!(d_L-k_1)^{n-i+1}} + (-k_1)^{n+1}\tfrac{\mathrm{e}^{-(d_L-k_1)t}}{(d_L-k_1)^{n+1}}\right).$$

The formula for L we calculated above is rather complex, so we decided to simplify it even more, assuming $k_1 << d_L$ and approximating

$$\tfrac{1-\mathrm{e}^{-(d_L-k_1)t}}{d_L-k_1} \approx \tfrac{1}{d_L}; \quad \tfrac{t^i}{i!(d_L-k_1)^{n-i+1}} \approx 0 \text{ for } i = 0, \dots n-1; \quad \tfrac{\mathrm{e}^{-(d_L-k_1)t}}{(d_L-k_1)^{n+1}} \approx 0$$

which yields

$$L \approx \frac{\alpha\,\mathrm{e}^{-k_1 t}}{d_L}\left(k_2 + \frac{k_1^{n+1}t^n}{n!}\right).$$

B Appendix. In this Appendix we present some results related to the analysis of System (8) and simplified System (10).

Let us focus on Eqs (8). Clearly, for any smooth decreasing positive function h_1 there exists unique solution of Eqs (8) which is positive for positive initial data. Below we show that solutions are bounded and there exists invariant subset of $(\mathbb{R}^+)^3$ for this system.

As h_1 is positive decreasing, we have $0 < h_1(z) \leq h_1(0)$. This implies

$$\dot{x} \leq h_1(0) - d_1 x \quad \Longrightarrow \quad x(t) \leq \max\left\{x(0), \tfrac{h_1(0)}{d_1}\right\} = x_{\max}.$$

Using this inequality we obtain

$$\dot{y} \leq p_2 x_{\max} - d_2 y \quad \Longrightarrow \quad y(t) \leq \max\left\{y(0), \tfrac{p_2 x_{\max}}{d_2}\right\} = y_{\max},$$

and eventually

$$\dot{z} \leq p_3 y_{\max} - d_3 z \quad \Longrightarrow \quad z(t) \leq \max\left\{z(0), \tfrac{p_3 y_{\max}}{d_3}\right\} = z_{\max}.$$

Having all the variables bounded from above we are in a position to show boundedness from below:

$$\begin{aligned}
\dot{x} \geq h_1(z_{max}) - d_1 x \quad &\Longrightarrow \quad x(t) \geq \min\left\{x(0), \tfrac{h_1(z_{\max})}{d_1}\right\} = x_{\min}, \\
\dot{y} \geq p_2 x_{\min} - d_2 y \quad &\Longrightarrow \quad y(t) \geq \min\left\{y(0), \tfrac{p_2 x_{\min}}{d_2}\right\} = y_{\min}, \\
\dot{z} \geq p_3 y_{\min} - d_3 z \quad &\Longrightarrow \quad z(t) \geq \min\left\{z(0), \tfrac{p_3 y_{\min}}{d_3}\right\} = z_{\min}.
\end{aligned}$$

Let $x_M = \frac{h_1(0)}{d_1}$, $y_M = \frac{p_2 x_M}{d_2}$, $z_M = \frac{p_3 y_M}{d_3}$, $x_m = \frac{h_1(z_M)}{d_1}$, $y_m = \frac{p_2 x_m}{d_2}$ and $z_m = \frac{p_3 y_m}{d_3}$. We obtain the following conclusion.

Corollary 10. *System* (8) *is positively invariant with respect to the set* $\Omega_3 = [x_m, x_M] \times [y_m, y_M] \times [z_m, z_M] \subset (\mathbb{R}^+)^3$.

Now, we present the proof of Proposition 1.

Proof. Looking for stability of SS, we calculate a Jacobian matrix of System 8

$$\begin{pmatrix} -d_1 & 0 & h_1'(\bar{z}) \\ p_2 & -d_2 & 0 \\ 0 & p_3 & -d_3 \end{pmatrix}$$

obtaining the following characteristic equation

$$0 = \lambda^3 + \lambda^2(d_1 + d_2 + d_3) + \lambda(d_1 d_2 + d_2 d_3 + d_1 d_3) + d_1 d_2 d_3 + P p_2 p_3,$$

where $P = -h_1'(\bar{z}) > 0$. According to the Routh-Hurwitz Criterion, stability is guaranteed by the inequality

$$(d_1 + d_2 + d_3)(d_1 d_2 + d_2 d_3 + d_1 d_3) > d_1 d_2 d_3 + P p_2 p_3. \tag{23}$$

For h_1 of the form (9), we have

$$P = \frac{p_1 b_1}{(1 + b_1 \bar{z})^2}, \quad \bar{z}^2 + \frac{1}{b_1}\bar{z} - \frac{p}{b_1 d} = 0 \Rightarrow \bar{z} = \frac{\sqrt{\Delta}}{2 b_1 d} - \frac{1}{2 b_1}, \ \Delta = d(d + 4 b_1 p),$$

where $d = d_1d_2d_3$ and $p = p_1p_2p_3$. Hence, Ineq (23) is equivalent to

$$(d_1 + d_2 + d_3)\left(\frac{1}{d_1} + \frac{1}{d_2} + \frac{1}{d_3}\right) > 1 + \frac{4b_1dp}{(d + \sqrt{d(d + 4b_1p)})^2}. \tag{24}$$

Note that $\frac{4b_1dp}{(d+\sqrt{d(d+4b_1p)})^2} < 1$, while $(d_1 + d_2 + d_3)\left(\frac{1}{d_1} + \frac{1}{d_2} + \frac{1}{d_3}\right) > 3$, which implies (24) and proves stability of SS. □

On the other hand, if P is large enough then the change of stability is possible. It is obvious that keeping all the parameters d_i on the fixed level and increasing the value of Pp_2p_3 we are able to violate (23). One of the possible options is to choose h_1 with a Hill coefficient γ greater than 1. Then for some threshold value γ_{th} of the bifurcation coefficient γ we obtain a pair of purely imaginary eigenvalues $\lambda = \pm i\omega$ and crossing this threshold γ_{th} we observe a change from stable to oscillatory dynamics via a Hopf bifurcation.

Properties of simplified System (10) are similar to those of System (8). Using the same line of reasoning we are able to find an invariant subset of $(\mathbb{R}^+)^2$.

Corollary 11. *System* (10) *is positively invariant with respect to the set* $\Omega_2 = [x_m, x_M] \times [z_m, z_M] \subset (\mathbb{R}^+)^2$, *where* $x_M = \frac{h_1(0)}{d_1}$, $z_M = \frac{p_3x_M}{d_3}$, $x_m = \frac{h_1(z_M)}{d_1}$, $z_m = \frac{p_3x_m}{d_3}$.

Now, we present the proof Proposition 3 for Eqs (10).

Proof. It is easy to check that we have one SS $(\bar{x}, \bar{z})$ satisfying

$$\bar{x} = \frac{d_3}{p_3}\bar{z}; \quad h_1(\bar{z}) = d_1\bar{x} = \frac{d_1d_3}{p_3}\bar{z}.$$

This state is locally stable, because its Jacobian matrix

$$J(\bar{x}, \bar{y}) = \begin{pmatrix} -d_1 & h_1'(\bar{z}) \\ p_3 & -d_3 \end{pmatrix}$$

has negative trace $-(d_1 + d_3)$ and positive determinant $d_1d_3 + p_3|h_1'(\bar{z})|$.

Global stability could be easily proved using the Dulac-Bendixson Criterion. Clearly, it is enough to take a divergence of the right-hand side of (10):

$$\frac{\partial}{\partial x}(h_1(z) - d_1x) + \frac{\partial}{\partial z}(p_3x - d_3z) = -(d_1 + d_3) < 0.$$

This shows that there is no periodic orbits of (10). All solutions are bounded and there is only one steady state which is locally stable, so it must be globally stable. □

C Appendix. Now, we focus on the properties of System (12) for constant drug amount $L(t) \equiv L \geq 0$, comparing its dynamics for $L = 0$ with the dynamics of System (10).

Proposition 12. *System* (12) *is positively invariant with respect to the set* $\Omega_L = [x_{mL}, x_M] \times [z_{mL}, z_{ML}] \subset (\mathbb{R}^+)^2$, *where* $x_M = \frac{h_1(0)}{d_1}$, $z_{ML} = \frac{h_3(x_M,L)}{d_3}$, $x_{mL} = \frac{h_1(z_{ML})}{d_1}$, $z_{mL} = \frac{h_1(z_{mL})}{d_3}$.

Proof. The proof is exactly the same as for System (8). □

Note that for $L = 0$ and the functions h_1, h_3 described by Eqs (9) and (11), respectively, the values x_{m0}, z_{m0}, z_{M0} tends with $b_3 \to 0$ to the respective values for System (10). Moreover, it is asy to see that $z_{M0} < z_M$, $x_{m0} > x_m$, while the relation between z_m and z_{m0} depends on the model parameters.

Now, we prove Proposition 4.

Proof. Any steady state SS $(\bar{x}_L, \bar{z}_L)$ of (12) satisfies

$$\bar{x}_L = \frac{h_1(\bar{z}_L)}{d_1}, \quad h_3\left(\frac{h_1(\bar{z}_L)}{d_1}, L\right) = d_3 z_L,$$

and we see that the equation for $\bar{z}_L$ has one positive solution as its right-hand side is increasing from 0 to ∞, while the left-hand side is positive decreasing.

Global stability could be shown exactly in the same way as for System (10), using the divergence of the system in the Dulac-Bendixson Criterion. □

Note that for the functions (9), (11), when $L = 0$ and $b_3 \to 0$ we have $(\bar{x}_0, \bar{z}_0) \to (\bar{x}, \bar{z})$ such that the dynamics of both model is almost identical, even for not very small values of b_3; cf. Fig 8 where $b_3 = 1$.

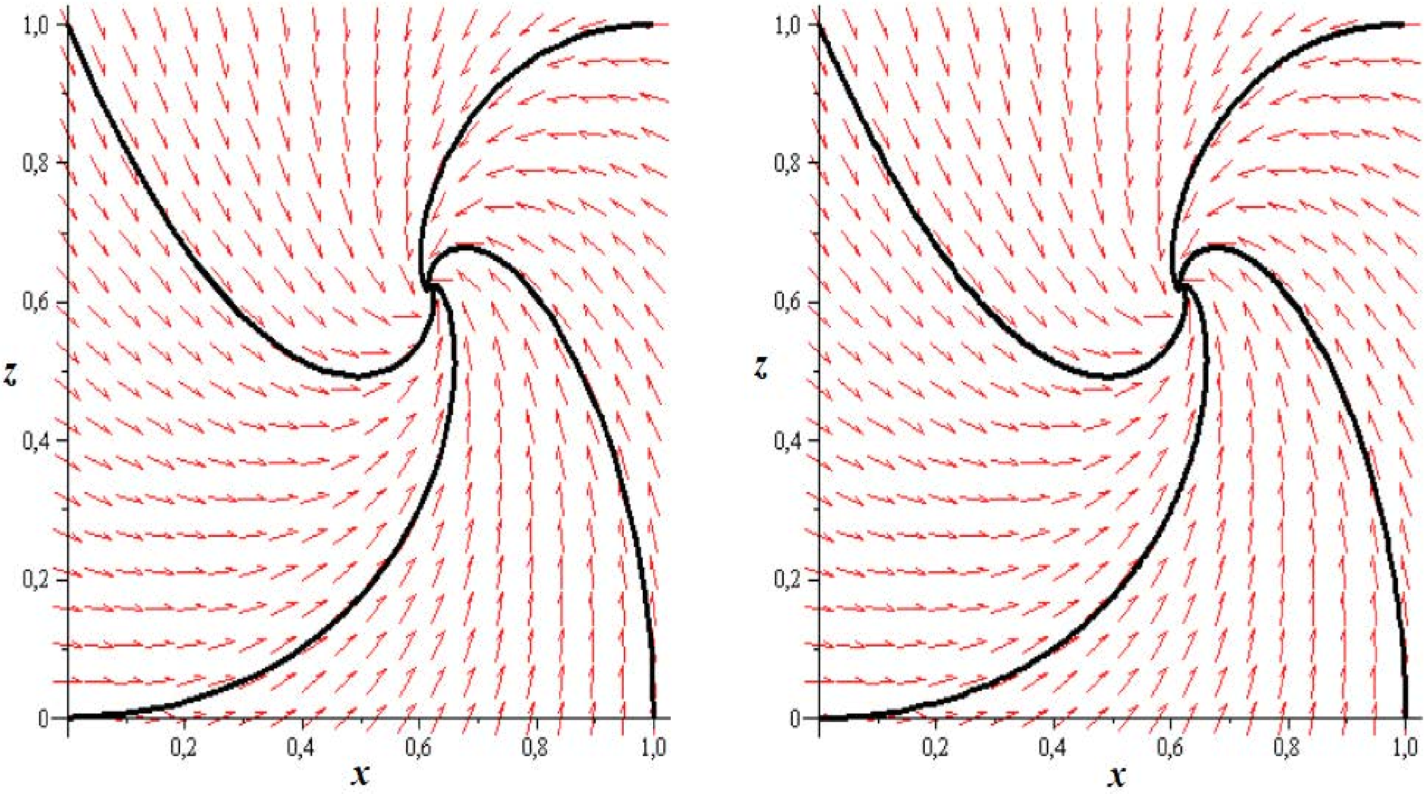

Fig 8. Examples of the phase space portraits for (10) (left) and (12) (right) with all parameters equal to 1.

D Appendix. Let us focus on the properties of Eq (14). Because this properties do not depend on the subscript i, we omit it to shorten the notation and consider

$$\dot{r} = \beta L \left(1 - \frac{r}{l}\right). \tag{25}$$

We are interested in the dynamics of solutions of Eq (25) mainly for constant amount of the drug. However, after a single dose of the drug the amount of it in the organism decreases, such that we can assume $\dot{L} \leq 0$.

Proposition 13. *For any continuous non-increasing positive function L there exists unique solution of Eq (25) which lies in the interval $(0, l]$ for initial data $r(0) \in [0, l]$.*

Proof. Existence of unique solution is obvious due to the properties of the right-hand side of Eq (25). Note that if $r(0) = 0$ then $\dot{r}(0) = \beta L$ and this value is positive after the drug application. This means that the solution starts to increase and it increases until $r(t) < l$. Assume that there exists first time-point $\tilde{t} > 0$ such that $r(\tilde{t}) = l$ and the solution exceeds l at this point, which means that $\dot{r}(\tilde{t}) > 0$ or $\dot{r}(\tilde{t}) = 0$ and $\tilde{t}$ is an inflection point. Clearly, we have

$$\dot{r}(\tilde{t}) = 0, \quad \ddot{r}(\tilde{t}) = \beta \left(\dot{L}(\tilde{t}) - \frac{L}{l}\right) < 0,$$

meaning that $\tilde{t}$ is not a point of inflection and the solution is not able to exceed the threshold l. It is also obvious that for $r(0) \geq 0$ we have $r(t) > 0$. □

Note that for any continuous function L we are able to solve Eq (25). In fact, it is enough to take integrable function L. We calculate

$$\int_{r(0)}^{r(t)} \frac{dr}{l - r} = \frac{\beta}{l} \int_0^t L(s)ds,$$

obtaining for $r(0) = 0$,

$$\ln |l - r|\Big|_0^{r(t)} = -\frac{\beta}{l} \int_0^t L(s)ds \quad \Longrightarrow \quad r(t) = l \left(1 - \exp\left(-\frac{\beta}{l} \int_0^t L(s)ds\right)\right).$$

Hence, for constant amount of the drug, $L(t) \equiv L$ we obtain

$$r(t) = l \left(1 - \mathrm{e}^{-\frac{\beta L}{l} t}\right),$$

while for any non-increasing function L,

$$r(t) \leq l \left(1 - \mathrm{e}^{-\frac{\beta L(0)}{l} t}\right),$$

which show that $r(t) \to r_g \leq l$ for $t \to \infty$, as r is monotonic.
